# DNA methylation remodeling and the functional implication during male gametogenesis in rice

**DOI:** 10.1186/s13059-024-03222-w

**Published:** 2024-04-02

**Authors:** Xue Li, Bo Zhu, Yue Lu, Feng Zhao, Qian Liu, Jiahao Wang, Miaomiao Ye, Siyuan Chen, Junwei Nie, Lizhong Xiong, Yu Zhao, Changyin Wu, Dao-Xiu Zhou

**Affiliations:** 1 National Key Laboratory of Crop Genetic Improvement, Hubei Hongshan Laboratory, Huazhong Agricultural University, Wuhan, 430070 China; 2 Key Laboratory of Plant Functional Genomics of the Ministry of Education/Jiangsu Key Laboratory of Crop Genomics and Molecular Breeding, College of Agriculture, Yangzhou University, Yangzhou, 225009 China; 3 Vazyme Biotech Co., Ltd, Nanjing, 210000 China; 4 Institute of Plant Science Paris-Saclay (IPS2), CNRS, INRAE, Université Paris-Saclay, 91405 Orsay, France

**Keywords:** Rice male gametogenesis, Egg, Zygote, DNA methylation, CMT3, JMJ706, JMJ707

## Abstract

**Background:**

Epigenetic marks are reprogrammed during sexual reproduction. In flowering plants, DNA methylation is only partially remodeled in the gametes and the zygote. However, the timing and functional significance of the remodeling during plant gametogenesis remain obscure.

**Results:**

Here we show that DNA methylation remodeling starts after male meiosis in rice, with non-CG methylation, particularly at CHG sites, being first enhanced in the microspore and subsequently decreased in sperm. Functional analysis of rice CHG methyltransferase genes CMT3a and CMT3b indicates that CMT3a functions as the major CHG methyltransferase in rice meiocyte, while CMT3b is responsible for the increase of CHG methylation in microspore. The function of the two histone demethylases JMJ706 and JMJ707 that remove H3K9me2 may contribute to the decreased CHG methylation in sperm. During male gametogenesis CMT3a mainly silences TE and TE-related genes while CMT3b is required for repression of genes encoding factors involved in transcriptional and translational activities. In addition, CMT3b functions to repress zygotic gene expression in egg and participates in establishing the zygotic epigenome upon fertilization.

**Conclusion:**

Collectively, the results indicate that DNA methylation is dynamically remodeled during male gametogenesis, distinguish the function of CMT3a and CMT3b in sex cells, and underpin the functional significance of DNA methylation remodeling during rice reproduction.

**Supplementary Information:**

The online version contains supplementary material available at 10.1186/s13059-024-03222-w.

## Background

DNA cytosine methylation is a hallmark for repression of transposable elements (TE) and related sequences in complex genomes such as flowering plants and vertebrates. In flowering plants, DNA cytosine methylation occurs in the context of CG, CHG and CHH (where H is A, C, or T) sequences. CG methylation is maintained during cell division by DNA methyltransferase1 (MET1), which recognizes and methylates hemi-methylated CG sites in the newly replicated DNA. Non-CG (i.e. CHG and CHH) methylation in heterochromatin is maintained by plant-specific Chromomethylases3 (CMT3, at CHG sites) and CMT2 (at CHH and CHG sites), which bind to the histone methylation mark H3K9me2, while CHH methylation in euchromatin regions is maintained by Domains Rearranged Methyltransferase2 (DRM2) guided by related siRNA [1]. DRM2 also mediates de novo DNA methylation regardless of sequence contexts [1]. All three methylation sites are found in TE and TE-like sequences and in about 10–20% of genes depending on plant species. DNA methylation in positive regulatory sequences impairs gene transcription and causes gene silencing, but enhances gene activity when occurring in repressive DNA elements [2]. CG methylation is also common within the transcribed regions of genes, where is associated with gene activity [3]. In rice, DNA methylation displays some differences in methylation levels and genomic distribution compared with Arabidopsis. For instance, rice mCHH is enriched at euchromatin regions and many genes are found to be methylated at CHH sites, especially around TSS [4, 5]. There are also quite a number of genes methylated at CHG sites which are related to allelic-specific expression in rice hybrids [6].

Epigenetic marks are reprogrammed in the gametes and after fertilization. In mammals, there are two distinct phases of epigenetic reprogramming to prevent inheritance of ancestral epigenetic signatures. The first phase consists of a genome-wide erasure of DNA methylation in the primordial germ cells (PGCs), the gamete precursors, followed by the reestablishment of epigenetic signatures to enable gamete maturation and function [7, 8]. Global DNA methylation is once again erased post-fertilization in early embryos, followed by another round of global de novo methylation during embryo development [9–11]. In contrast with mammals, DNA methylation is not globally erased in gametes in flowering plants [12–17]. Unlike mammalian germ lines that are defined already at an early stage of embryogenesis, thus before meiosis, the plant male and female sexual lineages initiate as diploid meiocytes from somatic cells, which give rise to haploid microspores after meiosis. The male microspores subsequently undergo mitosis, producing the vegetative and generative cells. The generative cell is further divided to produce two sperm cells in the mature pollen grain, the male gametophyte. Previous studies showed that plant sperm DNA methylation is remodeled and shows variation relative to somatic tissues [12, 14, 16–19]. However, it remains unclear whether the remodeling process is initiated in male meiocytes before meiosis or at the subsequent steps after meiosis and whether DNA methylation remodeling is required for male gametogenesis.

In this work, we analyzed DNA methylation in rice male meiocyte, microspore and sperm and studied the function of a set of chromatin regulators during the process. The results indicate that DNA methylation remodeling starts after meiosis of the male meiocytes and that non-CG methylation, particularly at CHG sequences, is dynamically remodeled during rice male gametogenesis. The work reveals distinct function of DNA methyltransferases and histone demethylase in the remodeling of CHG methylation and suggests that the CHG methylation remodeling has functional significance for male gametogenesis and fertilization.

## Results

### Non-CG methylation is dynamically remodeled during rice male gametogenesis

**Fig. 1:**
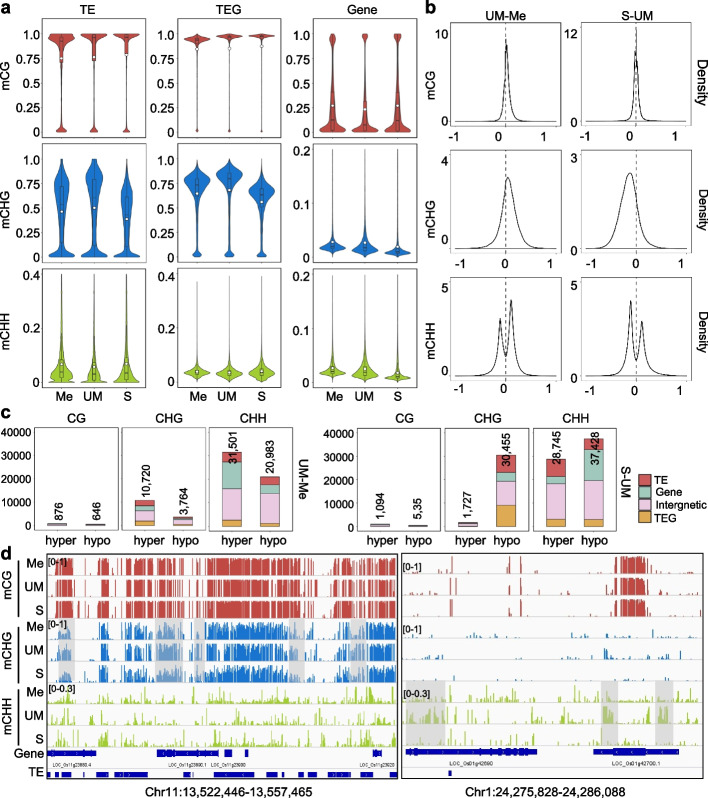
DNA cytosine methylation in rice meiocytes, unicellular microspores, sperms. **a** Violin plots showing overall cytosine methylation levels (mCG, mCHG, and mCHH) in transposable elements (TE), transposable gene (TEG) and protein coding gene (Gene) of rice Zhonghua 11 (ZH11) meiocyte (Me), unicellular microspore (UM) and sperm (S). Values of the methylomes are averages from the two replicates. The average methylation levels (white dots) and median values (black bars) are indicated. **b** Density plot showing the frequency distribution of fractional methylation difference between the indicated samples. **c** Numbers of differentially methylated regions (DMRs) of between the indicated comparisons, distributed in TE (> 500 bp), TEG, gene, and intergenic regions. DMRs located in TE (red), gene (light green), intergenic region (pink), and TEG (yellow) are shown. **d** Genome browser screenshots of mCG, mCHG, and mCHH in meiocytes (Me), unicellular microspore (UM), sperm (S). Differentially methylated regions are grey colored

To study DNA methylation dynamics during male gametogenesis in rice, we manually isolated male meiocytes, unicellular microspores and sperms of the Zhonghua11 (ZH11) variety as previously described [17, 20–22]. About 400 meiocytes, 300 unicellular microspores and 100 sperm cells were collected for bisulfite sequencing (BS-seq) analysis using a protocol developed for small numbers of cells (Additional file 1: Fig. S1a, b) [23]. Data with two biological replicates were obtained (Additional file 1: Fig. S1c, Additional file 2: Table S1). Violin plots of the BS-seq data revealed that the overall CG methylation (mCG) levels (especially in TE and TE-related genes, TEG) gradually increased during male gametogenesis (Fig. 1a), whereas CHG methylation (mCHG) levels were first increased in unicellular microspore (UM) but subsequently decreased in sperm (S) to the lowest levels (Fig. 1a). Density plots confirmed the mCHG variations between microspore (UM) and meiocyte (Me) and between sperm (S) and microspores (UM) (Fig. 1b). A similar trend of CHH methylation (mCHH) variation was also observed (Fig. 1b). Scanning of differentially methylated regions (DMRs, defined within 100-bp windows, see Methods) between microspore and meiocyte (UM-Me) detected more hyper than hypo CG (876 hyper versus 646 hypo), CHG (10,720 hyper versus 3,764 hypo), and CHH (31,501 hyper versus 20,983 hypo) DMRs, indicating a clear gain of non-CG methylation in microspore (Fig. 1c, d). In sperm relative to microspore, there were more hypo- than hyper-DMRs at non-CG, especially CHG context (30,455 hypo- compared to 1,727 hyper-DMRs), confirming a clear decrease of mCHG in sperm. The mCHG levels in sperm were the lowest when compared with those in egg and zygote or somatic tissues of the same rice variety (Additional file 1: Fig. S2a, see below), which could be also observed in other rice varieties (Additional file 1: Fig. S2b) [4, 16, 17, 24]. Analysis of sex cell methylomes obtained from the Nipponbare (NIP) variety indicated that sperm mCHG and mCHH levels were lower than pollen vegetative cell levels, but comparable to central cells of the female gametophyte (Additional file 1: Fig. S2b) [16, 24]. About 2/3 (5,221/7,775) of the increased mCHG (hyper DMRs) in microspore (relative to meiocyte) were erased in sperm, the other 1/3 (2,554/7,775) microspore hyper DMRs remained hyper-methylated in sperm (Additional file 1: Fig. S3a, clusters A and B). These two clusters of the microspore hyper DMRs were located mainly in genic and intergenic regions (Additional file 1: Fig. S3b), and showed enrichment of euchromatin histone marks (H3K36me3, H3K9ac) (Additional file 1: Fig. S3d). By contrast, many sperm hypo DMRs (12,588) that showed no change between microspore and meiocyte (Additional file 1: Fig. S3a), which corresponded mainly to TE and TEG (Additional file 1: Fig. S3b), and were enriched for the heterochromatin mark H3K9me2 (Additional file 1: Fig. S3d). The data indicate that during male gametogenesis mCHG and mCHH at both euchromatin and heterochromatin loci are dynamically remodeled to the lowest levels in sperm.

From cluster A of the microspore hyper DMRs, 315 genes were identified. These genes are enriched for translation, ribonucleoprotein complex, and cellular protein metabolic functions, implying that mCHG in protein translation and RNA-binding pathway genes was particularly dynamic during meiocyte to sperm development (Additional file 1: Fig. S3e, Additional file 4: Table S3). Several genes showed lower expression in microspore than meiocyte and/or sperm (Additional file 1: Fig. S4a), suggesting that hypermethylation might play a role in their repression in microspore.

### CMT3a and CMT3b function during male gametogenesis

**Fig. 2:**
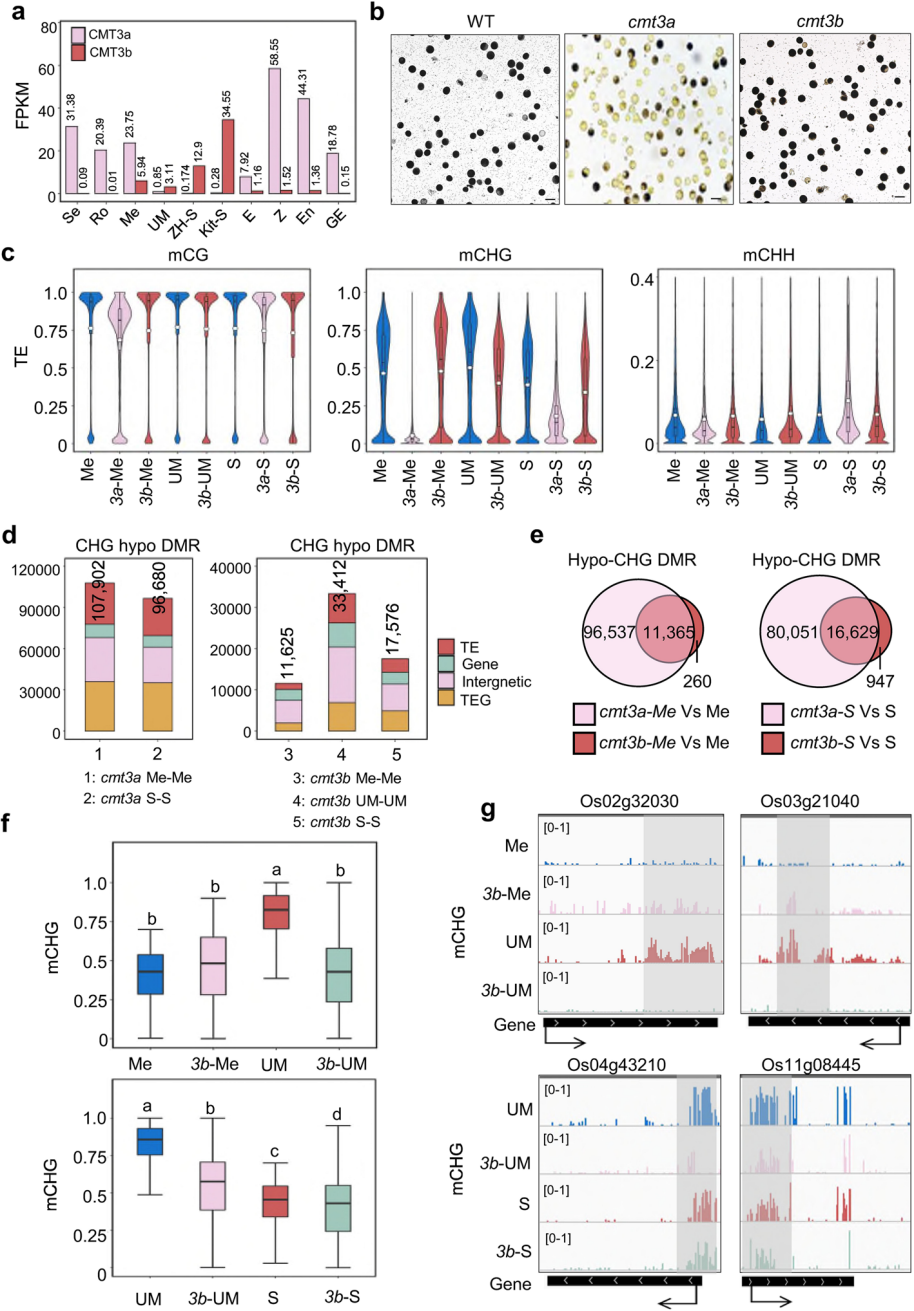
Effects of *cmt3a* and *cmt3b* mutations on DNA methylation in meiocyte, microspore and sperm. **a** Transcript levels in FPKM of rice CMT3a and CMT3b in seedling (Se), roots (Ro), meiocyte (Me), unicellular microspore (UM), sperm (S), egg (E), zygote (Z), endosperm nuclei (En, 1.5 days after fertilization) and globular embryo (GE, 3 days after fertilization) from RNA-seq data. The sperm (Kit-S) in Kitaake background was reported by Anderson et al., (2013). **b** The pollen grains of wild type and *cmt3a* and *cmt3b* mutants were I2-KI stained. Bars = 50 μm. **c** Violin plots comparing overall cytosine methylation levels of wild type and *cmt3a* and *cmt3b* mutant meiocyte (Me), unicellular microspore (UM) and sperm (S). The average methylation levels (white dots) and median values (black bars) in transposable elements (TE) are shown. Values of the methylomes are averages from the two replicates. **d** Number of differential methylated regions (DMR) in *cmt3a* and *cmt3b* relative to wild type. Relative portions in TE (> 500 bp), TEG, gene, and Intergenic regions are indicated by different colors. **e** Venn diagrams showing overlapping of hypo-CHG DMRs in *cmt3a* and *cmt3b* meiocyte (left) and sperm (right) relative to wild type cells. **f** Box plots of DNA methylation levels of hypo-CHG DMRs in meiocyte (Me) versus microspore (UM) (upper) and sperm (S) relative to microspore (UM) (lower) in wild type, *cmt3a* (3a) and *cmt3b* (3b) cells. The significance was calculated with multiple comparison tests. Different letters on top of the bars indicate a significant difference (*p* < 0.05). **g** Genome Browser screen captures showing high CHG methylation sites in microspore relative to meiocyte and sperm decreased in cmt3b mutants (highlighted by grey)

*CMT3a* is a major CHG methyltransferase gene expressed at high levels throughout the sporophytic development in rice [25, 26]. However, its expression became low or undetectable in sperm in several rice varieties (Fig. 2a). By contrast, *CMT3b* expression was low or undetectable in vegetative tissues/organs but showed expression in reproductive cells including meiocyte, microspore, and sperm (Fig. 2a). To study the function of *CMT3* genes during male gametogenesis, we produced *cmt3a* and *cmt3b* knockout (KO) plants in the ZH11 background using the CRISPR technique and two independent lines for each gene were obtained (Additional file 1: Fig. S5a). The *cmt3a* mutants produced mainly defective pollens and were infertile (Fig. 2b). Cytology sections revealed that the *cmt3a* pollen development was arrested likely at the bicellular microspore stage (Additional file 1: Fig. S5b).

By contrast, the *cmt3b* mutants showed no clear plant morphological phenotype. To check the effects of *cmt3* mutations on DNA methylation during male gametogenesis, we performed BS-seq of meiocyte, unicellular microspore, and sperm isolated from two independent CRISPR/Cas9-free lines of *cmt3a* and/or *cmt3b* at T3-4 generation (as well as a tissue culture-regenerated wild type line) (Additional file 2: Table S1). Violin plots of the data revealed that mCHG was almost absent from *cmt3a* meiocyte (Fig. 2c, Additional file 1: Fig. S6a), consistent with the *CMT3a* loss-of-function effects in somatic tissues [25]. To a lesser extent, mCG was also reduced in *cmt3a* meiocyte. However, *cmt3a* sperm mCHG (as well as mCHH) levels became higher than the mutant meiocyte (Fig. 2c), suggesting that additional activities partially restored mCHG and/or ectopically mediated mCHH in the mutant sperm. DRM2 and CMT2 being highly expressed in rice sperm (Additional file 1: Fig. S7a), the residual mCHG level in *cmt3a* sperm could be maintained by RdDM or CMT2. This hypothesis is supported by the observation that the *drm2/cmt2* mutations also reduced the mCHG levels of those loci in leaves (Additional file 1: Fig. S7b).

By contrast, the *cmt3b* mutation led to a clear loss of overall mCHG in microspore and sperm but the mutation effect was less clear in meiocyte (Fig. 2c, Additional file 1: Fig. S6a). The *cmt3b* mutation also resulted in some increases of mCHH in sperm. Density plots confirmed the observations (Additional file 1: Fig. S6b). The increases of mCHH in *cmt3a/b* sperm might be of indirect effects to compensate mCHG loss in the mutants. Nearly all of the *cmt3b* hypo-CHG DMRs in meiocyte and sperm overlapped with those of *cmt3a* (Fig. 2d, e), indicating that CMT3b functioned to maintain mCHG on a fraction of the CMT3a targets. The *cmt3b* mutation resulted in a large number (33,412) of hypo-CHG DMRs in microspore (Fig. 2d), and diminished the mCHG difference between microspore and sperm observed in wild type (Additional file 1: Fig. S6c). In fact, the methylation levels of hyper-CHG DMRs in wild type microspore versus meiocyte were decreased to the meiocyte levels in *cmt3b* microspore, and the methylation levels of the hypo-CHG DMRs in wild type sperm versus microspore were decreased to the sperm levels in *cmt3b* microspore (Fig. 2f, g). For the three clusters of DMRs shown in Additional file 1: Fig. S3a, the *cmt3b* mutation largely reduced the mCHG levels in microspore (Additional file 1: Fig. S3c). In addition, the 315 genes (from cluster A) showed higher mCHG in exons than introns in microspore (Additional file 1: Fig. S4b), while the *cmt3b* mutation reduced the mCHG levels from both exons and introns, suggesting that CMT3b may preferentially target gene exons in microspore (Additional file 1: Fig. S4a, b). The analysis indicates that *CMT3b* is required for the increase of mCHG in microspore.

### Histone demethylases JMJ706 and JMJ707 reduce CHG methylation

**Fig. 3:**
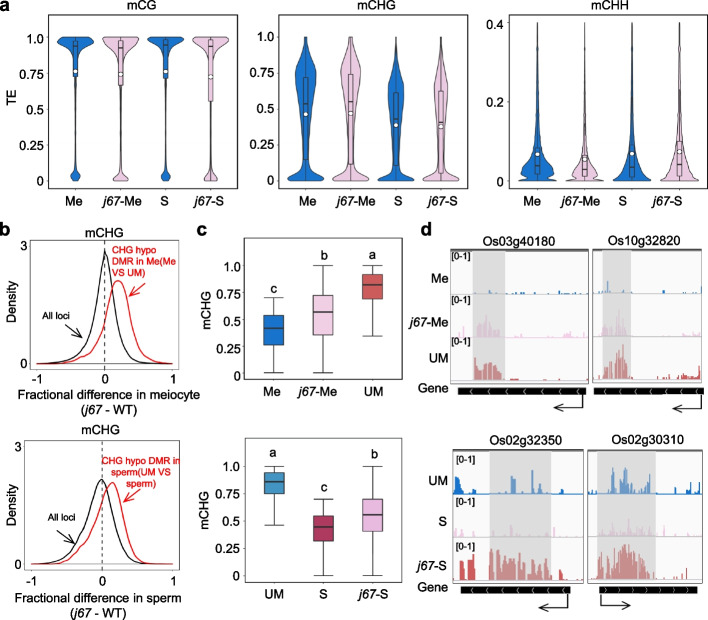
Effects of *jmj706/707* mutations on DNA methylation in male sex cells. **a** Comparison of overall TE methylation levels in wild type and *jmj706/707* mutant meiocyte (Me) and sperm (S). Values of the methylomes are averages from the two replicates. The average methylation levels (white dots) and median values (black bars) are shown. **b** Density plots of CHG methylation differences between *jmj706/707* mutant and wild-type meiocyte (upper) and sperm (lower) (black lines). The red traces are density plots confined to the hypo DMRs between meiocyte and microspore (Me-UM) (upper) or between sperm and microspore (S-UM) (lower). **c** Box plots showing DNA methylation levels of 50-bp hypo-CHG methylation regions in wild type meiocyte (Me) (upper) and sperm (S) (lower) relative to microspore (UM) and in *jmj706/707* meiocyte (j67-Me) and sperm (j67-S). The significance was calculated with multiple comparison tests. Different letters on top of the bars indicate a significant difference (p < 0.05). **d** Genome browser screen shots showing low CHG methylation sites in meiocyte (upper) or sperm (lower) relative to microspore but elevated in *j67* mutants. Grey illustrates differentially methylated regions

As *CMT3a* is not or lowly expressed in sperm, mCHG diluted by mitosis may not be maintained in sperm. Alternatively, active DNA demethylation may be involved, as mutations of DNA demethylases locally remodeled DNA methylation in sperm [17]. Since mCHG is linked to H3K9me2 through a positive feedback loop [27, 28], we investigated whether H3K9me2 demethylases were also involved in the decease of mCHG in sperm. JMJ706 was shown to function as a H3K9 demethylase in rice [29]. JMJ707 is closely related to JMJ706 [29], of which JMJ707 showed high expression in sperm (Additional file 1: Fig. S8a). To test whether the genes were involved in mCHG during male gametogenesis, we produced *jmj706* and *jmj707* double knockout (KO) plants and obtained two independent lines (Additional file 1: Fig. S8b). The KO lines (*j67*) showed a reduced pollen viability and seed setting rate (Additional file 1: Fig. S8c, d). We analyzed the DNA methylome of male meiocyte and sperm of the mutant lines (Cas9-free at T3-4 generation) and found that the mutations had no drastic effect on the overall methylation in the cells (Fig. 3a, Additional file 1: Fig. S9a). However, the methylation levels of the hypo-DMRs in wild type meiocyte versus microspore were increased in *j67* meiocyte (Fig. 3b, c, d). Similarly, the methylations levels of the hypo- DMRs in wild type sperm versus microspore were augmented in *j67* sperm (Fig. 3b, c, d). The *jmj706/7* mutations clearly elevated mCHG levels of cluster B DMRs in meiocyte and cluster C in sperm (Additional file 1: Fig. S3c). There was no overlap between *cmt3b* and *jmj706/7*-affected DMRs (Additional file 1: Fig. S9b). The analysis indicated that JMJ706/707 play a role to reduce mCHG at a set of genomic loci (mainly TE or TEG) in male meiocyte and sperm.

### Effect of the *cmt3a/b* and *jmj706/7* mutations on sexual lineage-specific methylation

It is shown that Arabidopsis male sex cells show sexual-lineage-specific methylation (SLM) or sexual-lineage-hypermethylation (SLH) [18]. Using the published methods [18], we identified 555 SLH loci in rice meiocyte, microspore and sperm relative to somatic cells (seedling) (Additional file 1: Fig. S10a). The *cmt3b* mutations reduced the SLH levels in all three male sex cell types, especially in microspore (Additional file 1: Fig. S10b, c). By contrast, the *jmj706/7* had no clear effect on SLH in the sex cells (Additional file 1: Fig. S10b). Further analysis could divide the 555 SLH into 340 canonical SLH and 215 SLM loci (Additional file 1: Fig. S11a) [18]. The canonical SLH loci corresponded mainly to TEs, while the SLM loci were located mainly in genes (body and promoter regions) (Additional file 1: Fig. S11b), suggesting that SLM mainly targets genic regions during male gametophyte and sperm development. In total, 132 genes were targeted by SLM, which are enriched for translational function (Additional file 1: Fig. S11c, Additional file 5: Table S4). The 132 genes appeared to be repressed in sperm compared to meiocyte or microspore (Additional file 1: Fig. S11d, e). The *cmt3b* mutation reduced SLM and increased expression of some of the genes in sperm (Additional file 1: Fig. S11d, e), suggesting that CMT3b may be involved in SLM and repression of some of the genes in sperm.

### Function of CMT3a and CMT3b in egg and zygote DNA methylation

**Fig. 4:**
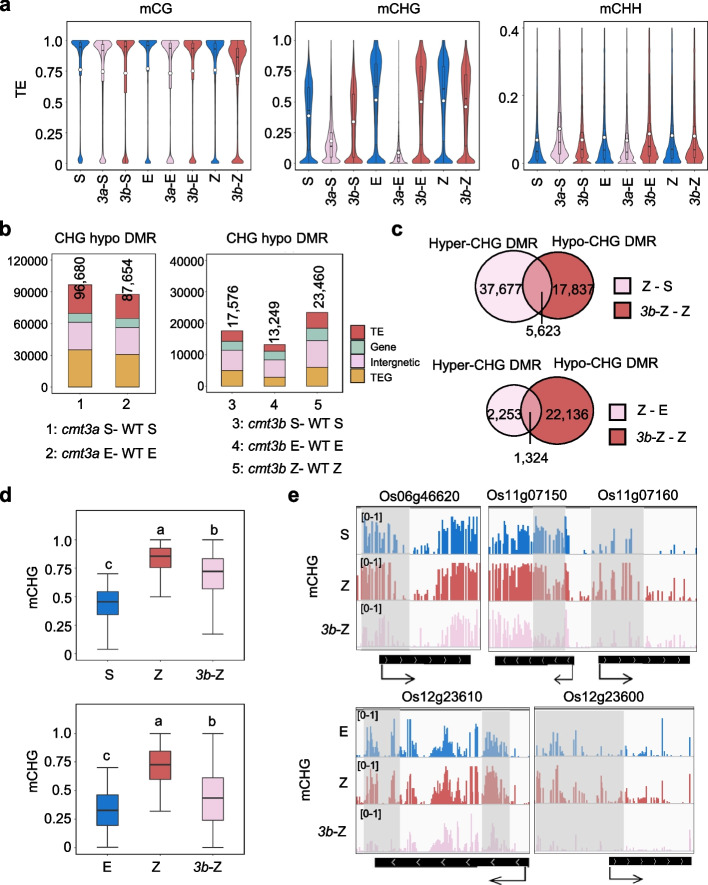
Effect of *cmt3a* and *cmt3b* mutations on DNA methylation in zygote and/or egg cells. **a** Comparison of overall TE methylation levels in sperm (S), egg (E) and zygote (Z) of wild type and *cmt3a, cmt3b* mutants. Values of the methylomes are averages from the two replicates. The average methylation levels (white dots) and median values (black bars) are shown. **b** Number of differential methylated regions (DMR) in the indicated mutant cells relative to wild type. Different colors indicate the distribution of DMR in TE (red), gene (light green), intergenic region (pink), and TEG (yellow). **c** Venn diagrams showing overlapping of hyper-CHG DMRs in zygote relative to sperm (Z-S) (upper) or egg (Z-E) (lower) and hypo-CHG DMRs in *cmt3b* zygote relative to wild type zygote (3bZ-Z). **d** Box plots showing DNA methylation levels of Z-S (upper) or Z-E (lower) hyper-CHG DMR of the indicated cell type. The significance was calculated with multiple comparison tests. Different letters on top of the bars indicate a significant difference (*p* < 0.05). **e** Screenshots of CHG methylation levels of 5 representative genes in the indicated cell

Unlike in sperm, *CMT3a* is highly expressed in egg and zygote. *CMT3b* transcripts are detected in Egg and zygote (Fig. 2a). To study *CMT3* function in egg and zygote, we compared wild type, *cmt3a* and/or *cmt3b* egg and zygote methylomes by BS-seq analysis (Additional file 2: Table S1). In wild type, egg and zygote mCHG levels were higher than sperm (Fig. 4a, Additional file 1: Fig. S12a). Because *cmt3a* was infertile, we only analyzed *cmt3a* egg methylome. As in male meiocyte and somatic tissues [25], the *cmt3a* mutation eliminated almost all mCHG in egg (Fig. 2c and Fig. 4a, Additional file 1: Fig. S12a). The *cmt3b* mutation had a limited effect on overall mCHG in egg, but caused a clear decrease of mCHG in zygote (Fig. 4a, Additional file 1: Fig. S12a). The *cmt3b* mutation produced more hypo-CHG DMRs (23,460) in zygote than egg (13,249) or sperm (17,576) (Fig. 4b). About 24% (5,623/23,460) of the hypo-DMRs in *cmt3b* zygote overlapped with the hyper-DMRs in wild type zygote versus sperm (Fig. 4c). In fact, the *cmt3b* mutation reduced the methylation differences of the DMRs between wild type zygote and sperm (Z-S) or egg (Z-E) (Fig. 4d, e). Together, the data indicate that CMT3b participates in reestablishing mCHG methylation at a subset of the Z-S and Z-E DMRs in the zygote.

### Function of rice CMT3b in gene expression in reproductive cells

**Fig. 5:**
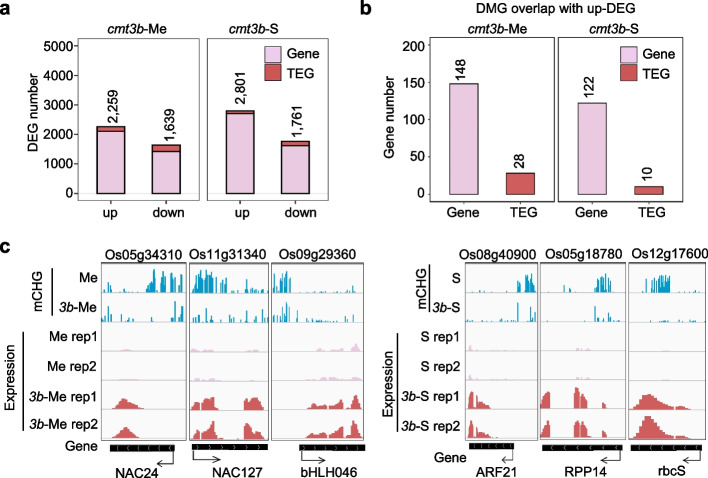
The *cmt3b* mutation affected non-TE gene expression in meiocyte and sperm.**a** Number of differentially expressed non-TE genes (pink) and TE-related genes (TEG) (red) in *cmt3b* mutant meiocyte and sperm relative to wild type (padj < 0.01, FC > 2). The highly expressed genes (TPM > 10) in sperm were filtrated for comparison. **b** Number of upregulated genes overlapping with hypo-CHG methylated genes (DMG) detected in *cmt3b* meiocyte and sperm relative to the wild type cells. **c** Genome browser screenshots of the methylation and expression levels of representative genes of the 148 (left) and 122 (right) genes shown in (**b**)

To study the *cmt3* mutation effects on gene expression in sex cells, we first performed RNA-seq of wild type and *cmt3b* male meiocyte and sperm. Three replicates (two replicates for WT sperm) were performed (Additional file 1: Fig. S13a, Additional file 3: Table S2). PCA analysis indicated a high reproducibility of the replicates (Additional file 1: Fig. S13b). The WT meiocyte transcriptome showed high correlation with previously published rice meiocyte transcriptomes (*r* > 0.85) (Additional file 1: Fig. S13c). In *cmt3b* meiocyte, 2,259 and 1,639 genes were respectively up- and downregulated (Fig. 5a). Similar numbers of differentially expressed genes (DEGs) were detected in the mutant sperm (Fig. 5a). Upregulated genes in *cmt3b* meiocyte were enriched for gene transcription function, while upregulated genes in *cmt3b* sperm were mainly enriched for translational function (Additional file 1: Fig. S14a, b). A small number of upregulated DEGs were found to associate with hypo-DMRs in the mutant cells (Fig. 5b, c; Additional file 6: Table S5). The analysis suggests that CMT3b plays a role in shutdown of transcriptional activity in meiocyte for preparation of meiosis and may contribute to the low translational activity in sperm [30].

In parallel, we analyzed the egg and zygote transcriptomes of wild type and *cmt3a/b* plants. Because *cmt3a* was infertile, we only analyzed *cmt3a* egg transcriptome. PCA analysis indicated high levels of reproducibility of the replicates. The *cmt3b* egg and zygote transcriptomes were close to, but distinct from, the wild type (Additional file 1: Fig. S13b). By contrast, the *cmt3a* egg transcriptome was largely distal from the wild type (Additional file 1: Fig. S13b), consistent with the drastic effect of *cmt3a* mutation on mCHG in egg. There were in total 4,868 upregulated genes (> two-fold, Q < 0.01), of which 1,648 were hypomethylated at CHG sites in *cmt3a* egg (Additional file 1: Fig. S12b, c). Interestingly, among the up-regulated genes, 1,461 were TEGs, of which 982 (67.2%) were hypomethylated at CHG sites in *cmt3a* egg (Additional file 1: Fig. S12b, c). The analysis indicates that CMT3a-mediated mCHG is required mainly for TEGs repression in egg, consistent with previous results showing that *cmt3a* mutation resulted in burst of TE expression [26]. The *cmt3b* mutation resulted in totally 3,022 upregulated genes (> two-fold, Q < 0.01) in egg, of which few were TEGs and only 120 (including 26 TEG) were identified as hypo-CHG methylated genes in the mutant egg (Additional file 1: Fig. S12b, c), indicating that unlike *cmt3a*, the *cmt3b* mutation de-repressed mainly non-TE-related genes, which was likely independent of a clear loss of mCHG. However, about 40% of the upregulated genes in *cmt3b* overlapped with those detected in *cmt3a* eggs (Additional file 1: Fig. S12d), suggesting that both CMT3 genes are required for gene (mainly non-TEGs) repression in egg. The *cmt3b* mutation resulted in upregulation of 2,179 and downregulation of 1,870 genes in zygote (Additional file 1: Fig. S12b). Similar to that observed in *cmt3b* egg, relatively few upregulated genes were TEGs or showed hypo mCHG in *cmt3b* zygote (Additional file 1: Fig. S12c).

### CMT3b represses zygotic genes in egg cells

**Fig. 6:**
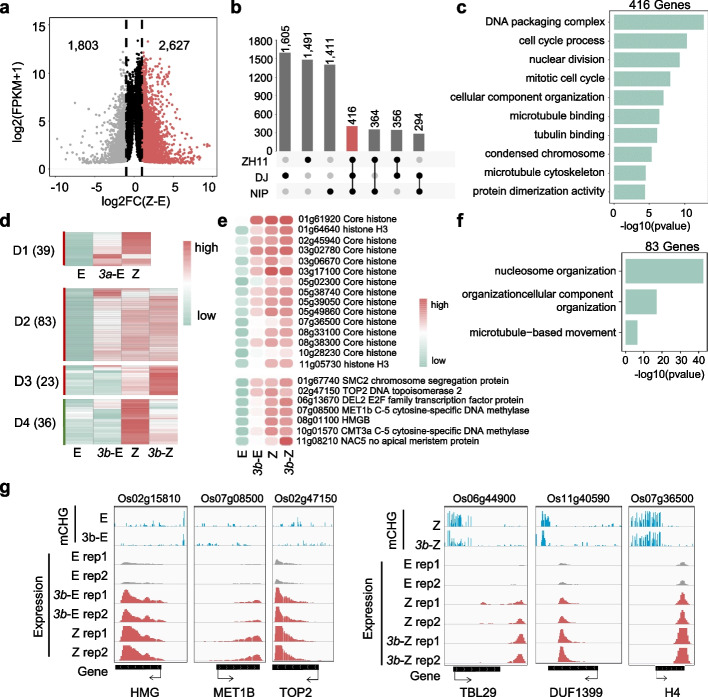
The *cmt3b* mutation de-represses zygote-expressed genes in egg. **a** Number of differentially expressed genes in wild type zygote relative to egg (FC > 2, *p* < 0.01). **b** Overlaps of high zygotic expression gene relative to egg in NIP, DJ and ZH11 varieties (Anderson et al., 2017; Zhou et al., 2021). **c** GO enrichment of zygote-expressed genes detected in three cultivars. **d** Transcript heatmaps of the 416 zygote-expressed genes in *cmt3b* egg (3b-E) and zygote (3b-Z) compared with wild type egg (E) and zygote (Z). **e** Transcript heatmaps of several representative zygote genes upregulated in *cmt3b* egg. **f** GO enrichment of the 83 upregulated genes in *cmt3b* egg shown in (**d**). **g** Integrative Genomics Viewer screenshots of six examples of the genes described in (**d**)

In wild type zygote we identified 1804 down- and 2628 upregulated (> two-fold, Q < 0.01) genes relative to egg (Fig. 6a). Among the upregulated genes, 416 overlapped with previously identified genes expressed in rice zygotes (Fig. 6b) [31]. The 416 genes were enriched for chromatin replication and cell division functions (Fig. 6c), consistent with zygotic genome activation to promote zygote cell division in plants [32]. Within the 416 zygotic genes, 83 were upregulated in *cmt3b* egg. By contrast, although the *cmt3a* mutation caused a larger number of upregulated genes in egg, only 39 were among the 416 zygotic genes (Fig. 6d). Among the 83 zygotic genes upregulated in *cmt3b* egg were those encode histones, chromatin proteins (HMGs, SMC2, TOP2), DNA methyltransferases (MET1 and CMT3a), transcription factors (E2F, HAP3, NAC), and cell division-related proteins (Fig. 6e, f, g, Additional file 7: Table S6). The analysis suggested that CMT3b has a function to repress zygote gene expression program in egg.

## Discussion

### Non-CG methylation dynamics during rice male gametogenesis

Previous results showed that DNA methylation in plants is partially remodeled or reconfigured in male and female gametes [12–17, 19]. In this work, we provided evidence that non-CG (mainly CHG) methylations are dynamically remodeled during rice male gametogenesis with the highest levels observed in microspore and the lowest levels in sperm. It is known that during male germline development there is a cell cycle arrest [33], which could account for the reduced mCHG and mCHH in sperm. Unlike in Arabidopsis meiocyte that has higher mCG but lower mCHH than in somatic tissues [18], DNA methylation levels in rice male meiocyte were similar to somatic tissues (Additional file 1: Fig. S15), suggesting that the remodeling process likely starts after meiosis in rice. Although the underlying function of the dynamic remodeling of mCHG (i.e. first increased in microspore then reduced in sperm) remains to be further explored, association of substantial numbers of genes with hypo mCHG in meiocyte and sperm relative to microspore of hundreds of genes (enriched for translational function) suggest that the remodeling is linked to gene expression dynamics during male gametogenesis. This is supported by the identification SLM, which targets mainly genes that are repressed in sperm. Alternatively, the remodeling may associate with chromatin changes during male gametogenesis.

The increase of mCHG observed in microspore was unexpected, as the methylation levels would be reduced after the meiotic cell divisions. The enhanced mCHG may have functional significance in microspore development. It remains to be studied whether the increased mCHG is related to chromatin reorganization of the haploid genome that was shown to be activated before the bicellular microspore stage in cereals [34]. As hyper-methylated genes are enriched for ribonucleoprotein complex (such as mRNA-binding) and translation proteins in microspore, the mCHG increase may be also related to repression of genes involved in transcriptional and translational activities that are shutdown during meiosis or male gametogenesis [26].

The decrease of mCHG in sperm may be required for sperm development and/or reshaping the sperm chromatin that lacks the compact silent center (CSC) found in egg and zygote chromatin 3D structures [21], and contains specific histone variants and modification patterns [35–39]. Alternatively, the decreased mCHG might facilitate the decondensation of the sperm chromatin upon fusion with the egg cell nucleus after fertilization, allowing initiation of transcription from the paternal genome [40].

The remodeling of non-CG methylation in rice sperm seemed to differ from Arabidopsis sperm where mCHH is lost or largely reduced [12]. This may be due to a different mCHH landscape in the rice genome, which is mainly scattered in genic regions [4, 5]. In fact, in rice sperm mCHH was found to be reduced in genic regions, but appeared to be enhanced in TE-rich pericentromeric regions (Additional file 1: Fig. S16a, b). The increased mCHH at pericentromeric region in sperm may be indirectly promoted by DNA demethylation at TE and repetitive sequence in the pollen vegetative cell in rice [16]. Alternatively, siRNAs produced by microspore may be inherited into sperm, in which they target heterochromatin LTR retrotransposon silencing through the RdDM pathway. In addition, the reduced genic mCHG and mCHH may also involve DNA demethylases that were shown to function in rice sperm [17].

### Distinct functions of CMT3a /b in reproductive cells

CMT3a is the major CHG methyltransferase in rice. *CMT3a* expression was detected in meiocyte, but barely in sperm (Fig. 2a), which may contribute to the low mCHG levels in sperm cells. However, CMT3a-mediated mCHG appeared essential for post-meiotic development, as the *cmt3a* mutation that eliminated mCHG in meiocyte stopped the pollen development at bicellular microspore stage. The expression pattern and mutation effects indicate that CMT3b plays an important role in increasing mCHG in the microspore in addition to complementing CMT3a for mCHG maintenance in meiocyte and sperm. However, the present data suggest that CMT3b preferentially targets protein-coding genes for methylation and is required for repression of transcriptional and/or translational genes during male gametogenesis.

The present work indicated CMT3a/b also have distinct functions in egg. CMT3a plays the major role in silencing TEGs, whereas CMT3b is required for repression of zygote gene expression program in egg. The repression of cell vision genes by CMT3b in egg is consistent with previous results showing that rice and maize egg cells are almost devoid of transcripts encoding histone proteins [31, 41]. The *cmt3b* effects on zygote mCHG and gene expression indicate that CMT3b participates in zygotic genome reprograming by reestablishing mCHG methylation and by regulating gene expression. In conclusion, the work reveals non-CG DNA methylation dynamics during male gametogenesis and distinguishes CMT3a/b functions in mCHG and gene expression in reproductive cells in rice.

## Conclusions

The work shows that during male gametogenesis mCHG level is first enhanced in microspores but subsequently reduced to the lowest level in sperm. CMT3a is the main CHG methyltransferase in somatic cells and meiocyte to silence TE and TE-related genes, while CMT3b is required for the surge of mCHG in microspore to repress transcription and translation-related genes, and is involved in the establishment of the zygotic epigenome after fertilization. The histone H3K9me2 demethylases JMJ706 and JMJ707 contribute to the reduction of mCHG in sperm. This study reveals a dynamic remodeling of mCHG during male gametogenesis, which has functional significance for pollen development and fertilization.

## Methods

### Plant materials and growth conditions

Rice variety Zhonghua11 (ZH11) (*Oryza sativa* spp. *japonica*) was used for transformation of *CMT3a*, *CMT3b*, and *JMJ706/707* CRISPR/Cas9 vectors in this study. Single-guide RNAs (sgRNA) of CRISPR/Cas9 system were designed as previously reported [42]. The sgRNA target sequences of *CMT3a*, *CMT3b*, and *JMJ706/707* as well as primers for genotyping are listed in Additional file 8: Table S7. Mutations in *CMT3a*, *CMT3b* and *JMJ706/707* were decoded by DSDecodeM (http://skl.scau.edu.cn/dsdecode/) [43, 44]. Cas9-free transgenic plants from T3-T4 segregating populations were utilized for phenotypic analysis and isolation of reproductive cells. For field growth, germinated rice seedlings were planted in Wuhan from May to October. For greenhouse growth, germinated rice seedlings were planted in soil-filled boxes under a 14-h light/10-h dark cycle at temperatures of 32 °C (in light) and 26 °C (in dark).

### Sperm, meiocyte and unicellular microspore isolation

Rice sperm, meiocyte and unicellular microspore were isolated from anthers of Cas9-free transgenic plants and tissue culture-regenerated wild type plants. For sperm isolation, mature anthers were soaked in 45% (w/v) sucrose and then transferred into 15% (w/v) sucrose to release sperm pairs. For isolation of meiocytes, panicles of about 5 cm length were chosen and middle parts of the panicles were collected. Intact anthers were carefully selected using dissecting needles, placed onto an RNAase-free slide, and suspended in a PBS solution containing 1% proteinase inhibitors. The outer wall of anthers was then removed by capillary to release meiocytes. Isolated meiocytes were checked under a light microscope (Additional file 1: Fig. S1a). For isolation of unicellular microspores, panicles of about 8 cm length were harvested and the middle parts of the panicles were collected. Anthers were dissected out and made a hole with a capillary tube in the same solution as used for meiocyte isolation. Squeeze gently from the opposite side of the hole to release unicellular microspore. Isolated microspores were checked under a light unicellular microscope (Additional file 1: Fig. S1b). All isolated cells were collected by a micromanipulator system (Eppendorf, TransferMan® 4r).

### Egg and unicellular zygote isolation

Rice eggs and unicellular zygotes were isolated from ovules before and after fertilization of Cas9-free transgenic plants and tissue culture-regenerated wild type [17, 21]. Briefly, ovaries of non-pollinated and pollinated florets (about 6.5 h after pollination) were manually collected in RNAase-free water under a dissection microscope. The collected ovules were transferred to a solution containing 0.53 M mannitol and 1% proteinases inhibitors to release eggs or zygotes. All isolated cells were stained by FDA (Fluoresceinc diacetate, Sangon, 596–09-8) and collected by a micromanipulator system (Eppendorf, TransferMan® 4r).

### RNA-seq and BS-seq library construction

For RNA-seq library construction, mRNA isolated from different types of cells were reverse transcribed and amplified by using a Single Cell Full Length mRNA Amplification Kit (Vazyme, Cat.# N712) according to manufacturer’s instruction. cDNAs were sheared into 200–400 bp DNA fragments followed by purification using VAHTS DNA Clean Beads (Vazyme, Cat.# N411). The rest steps were performed using the TruePrep DNA Library Prep Kit V2 for Illumina (Vazyme, Cat.# TD502). About 3000 sperm cells and 400 meiocytes were used to construct the transcriptome libraries. Fifty cells of egg or unicellular zygote were used for each replicate of the transcriptome library construction. BS-seq libraries were constructed using reported protocol [23]. About 300–400 cells were pooled for each replicate for sperm, meicoyte and unicellular microspore bisulfite seq library construction and fifty cells of egg or unicellular zygote were used to construct bisulfite seq library.

### Semi-thin section

Proper florets were gathered and 50% FAA (50 ml absolute ethanol, 10 ml 37% formaldehyde solution, 5 ml glacial acetic acid, add double distilled water to 100 ml) was used to fix samples. Semi-thin embedding were performed using Herau Kulzer Technovit 7100 resin. Briefly, the materials were first transferred to a gradient alcohol solution (70% for 4 h at 4° C, 85% for 1 h at room temperature, 95% for 1 h at room temperature, 100% for 2 h at room temperature), then immersed separately in a pre-infiltration solution (equal parts of 96% or absolute ethanol and base liquid Technovit 7100) and an infiltration solution (1 g hardener I dissolved in 100 ml base liquid) for two hours. The treated materials were embedded in a 65° C film stand with polymerisation solution (1 ml hardener is added with the help of a pipette and stirred into 15 ml of preparation solution) and then sliced.

### RNA-seq data analysis

First, raw RNA-seq data were cleaned using fastp software to remove connectors and filter low-quality reads [45]. Clean read was then matched to the MSU7.0 rice reference genome using hisat2 software [46]. FeatureCounts were used for quantitative analysis and the differentially expressed genes (Fold change > 2, q < 0.01) were calculated by DESeq2 [47].

### BS-seq data analysis

Fastp software was used to remove connectors and filter low-quality reads from the acquired raw BS-seq data. Clean reads were mapped to the MSU7.0 rice genome. Bismark software was used to match, deduplicate and extract methylation sites [48]. To obtain more loci for analysis, we combined the two biological replicates. Duplicates were removed and uniquely mapped reads were retained and each cytosine covered by at least five reads for further analysis. To avoid methylation being strongly influenced by single cytosine sites, the methylation level of each cytosine is calculated separately and then averaged for all cytosines to represent the methylation level for each bin (bin methylation level = (sum of individual cytosine methylations) / (number of cytosines within 100 bp).

To identify differential methylated regions (DMRs), the whole genome was divided into 100-bp bins. Bins that contained at least five cytosines each and every cytosine with at least a five-fold coverage were retained, absolute methylation difference of 0.5, 0.3, and 0.1 for CG, CHG, and CHH, respectively, and *P* values < 0.01 (Fisher’s exact test) were considered as DMRs. Neighboring DMRs within 200 bp were merged.

TE gene and gene Annotation information was downloaded from the Rice Genome Annotation project (http://rice.uga.edu/pub/data/Eukaryotic_Projects/o_sativa/annotation_dbs/pseudomolecules/version_7.0/all.dir/all.locus_brief_info.7.0). Differentially methylated genes (DMG) were identified using bedtools software [49], filtrated by > 80% of overlap between DMRs and genes.

Density plots show the frequency distribution of DNA methylation differences between 50-bp window of two samples with at least 20 informative sequenced cytosines in both samples and 70% CG, 30% CHG, or 10% CHH methylation in either of the samples as previously described [16].

### Identification of sexual lineage-specific methylation loci

The previously reported method [18] was used to identify SLH and SLM in rice male sex cells. Briefly, Average sex cell mCG and mCHH levels within 100 bp bins were calculated from meiocytes, microspores and sperm, and average sex cell mCHG levels were calculated from meiocytes and microspores. Fractional methylation in 100 bp windows across the genome was compared between an average of selected sex cells (SexAV) and somatic tissues (Seedling) (Diff = SexAV—Seedling). The total methylation level was significantly different (Fisher's exact test *p*-value < 0.01), and the methylation level of all sex cell replicates was higher than that of all somatic tissues and selected windows meeting the following criterion: Diff_CG > 0 & Diff_CHG > 0.05 & Diff_CHH > 0.1 & (Diff_CG + Diff_CHG + Diff_CHH) > 0.4.

The refined list of SLHs (555 loci) was then separated into two groups based on the level of CHH/G methylation in somatic tissues: 1) SLMs with CHH and CHG methylation lower than 0.05 and 0.1, respectively, in somatic tissues (215 loci). 2) canonical SLHs with CHH methylation higher than 0.05 or CHG methylation higher than 0.1, in somatic tissues (340 loci) [18].

### Supplementary Information

**Additional file 1: Figure S1.** Isolation of rice meiocytes and unicellular microspores and quality control of rice male germ cells BS-seq. **Figure S2.** DNA methylation levels in reproduction cells compared with somatic tissues. **Figure S3.** Analysis of microspore hyper DMRs relative to meiocyte and sperm. **Figure S4.** Analysis of mCHG genes in unicellular microspore. **Figure S5.** Effects of *cmt*3*a* and *cmt*3*b* in pollen development. **Figure S6.** CMT3b maintains high mCHG level in microspore. **Figure S7.** Analysis had no effect on mCHG in cmt3a mutant sperm cells. **Figure S8.** Production and phenotypic analysis of jmj706/707 double mutants. **Figure S9.** Average methylation level of TEG and genes in jmj706/707 meiocyte and sperm cells compared with wild type. **Figure S10.** Effect of the cmt3a/b and jmj706/7 mutations on sexual lineage-specific methylation. **Fig. S11.** Analyze the Canonical SLH and lineage-specific methylation (SLM). **Figure S12.** Effects of cmt3a and cmt3b mutations in zygote and/ or egg DNA methylation. **Figure S13.** Transcriptome data analysis. **Figure S14.** Enrichment of upregulated genes in *cmt3b* meiocyte and sperm. **Figure S15.** DNA methylation levels in rice reproductive cells and seedlings. **Figure S16.** DNA CHH methylation landscape in rice reproductive cells and seedlings.

**Additional file 2: Table S1.** Summary of BS-seq data.

**Additional file 3: Table S2.** Summary of RNA-seq data.

**Additional file 4: Table S3.** 315 genes annotated by Cluster A.

**Additional file 5: Table S4.** 132 genes annotated by SLM.

**Additional file 6: Table S5.** CMT3b regulates gene in meiocyte and sperm.

**Additional file 7: Table S6.** 83 zygotic genes upregulated in *cmt3b* egg.

**Additional file 8: Table S7.** Primers used in this study.

**Additional file 9. **Review history.

## Data Availability

Genes sequence data from this article can be found in the Rice Genome Annotation Project website under the following accession numbers: CMT3a, LOC_Os10g01570, CMT3b, LOC_Os03g12570, JMJ706, LOC_Os10g42690, JMJ707, LOC_Os02g46930. All high throughput data in support of the finding of this study deposited to the Gene Expression Omnibus (GEO) under the accession number GSE235680 [50]. The transcription profiles of reported rice sperm were downloaded from Gene Expression Omnibus (accession no. GSE50777) [51]. The rice central cells and vegetative cells BS-seq data was under the accession number GSE89789 [52] and GSE126791 [53]. The rice *cmt2* and *drm2* mutant BS-seq data was under the accession number GSE138705 [54]. No other scripts and software were used other than those mentioned in the Methods section.
