# Author Correction: DNA methylation remodeling and the functional implication during male gametogenesis in rice

**DOI:** 10.1186/s13059-024-03344-1

**Published:** 2024-07-27

**Authors:** Xue Li, Bo Zhu, Yue Lu, Feng Zhao, Qian Liu, Jiahao Wang, Miaomiao Ye, Siyuan Chen, Junwei Nie, Lizhong Xiong, Yu Zhao, Changyin Wu, Dao-Xiu Zhou

**Affiliations:** 1 National Key Laboratory of Crop Genetic Improvement, Hubei Hongshan Laboratory, Huazhong Agricultural University, Wuhan, 430070 China; 2 Key Laboratory of Plant Functional Genomics of the Ministry of Education/ Jiangsu Key Laboratory of Crop Genomics and Molecular Breeding, College of Agriculture, Yangzhou University, Yangzhou, 225009 China; 3 Vazyme Biotech Co., Ltd, Nanjing, 210000 China; 4 Institute of Plant Science Paris-Saclay (IPS2), CNRS, INRAE, Université Paris-Saclay, 91405 Orsay, France

**Author Correction: Genome Biol 25, 84 (2024)**

**https://doi.org/10.1186/s13059-024-03222-w**

Following publication of the original article [1], the authors identified an error in Fig. 2. In Fig. 2B, a wild type pollen picture was wrongly used to represent cmt3b pollens that in fact are of wild type phenotype.

The incorrect and correct Fig. 2 is published in this correction article and the original article [1] has been updated.

Incorrect figure:

**Fig. 2:**
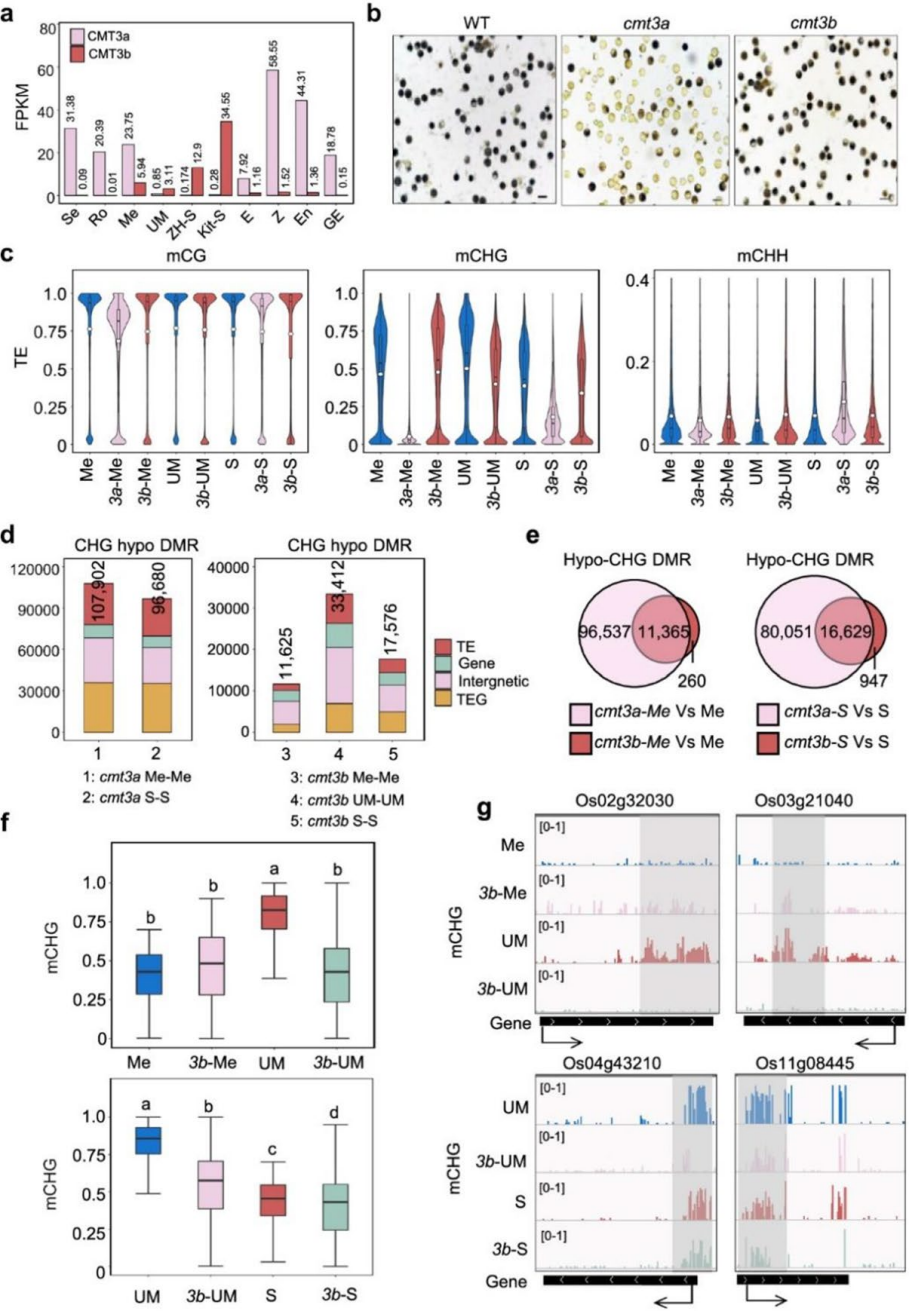
Effects of *cmt3a* and *cmt3b* mutations on DNA methylation in meiocyte, microspore and sperm. **a** Transcript levels in FPKM of rice CMT3a and CMT3b in seedling (Se), roots (Ro), meiocyte (Me), unicellular microspore (UM), sperm (S), egg (E), zygote (Z), endosperm nuclei (En, 1.5 days after fertilization) and globular embryo (GE, 3 days after fertilization) from RNA-seq data. The sperm (Kit-S) in Kitaake background was reported by Anderson et al., (2013). **b** The pollen grains of wild type and *cmt3a* and *cmt3b* mutants were I2-KI stained. Bars = 50 μm. **c** Violin plots comparing overall cytosine methylation levels of wild type and *cmt3a* and *cmt3b* mutant meiocyte (Me), unicellular microspore (UM) and sperm (S). The average methylation levels (white dots) and median values (black bars) in transposable elements (TE) are shown. Values of the methylomes are averages from the two replicates. **d** Number of differential methylated regions (DMR) in *cmt3a* and *cmt3b* relative to wild type. Relative portions in TE (> 500 bp), TEG, gene, and Intergenic regions are indicated by different colors. **e** Venn diagrams showing overlapping of hypo-CHG DMRs in *cmt3a* and *cmt3b* meiocyte (left) and sperm (right) relative to wild type cells. **f** Box plots of DNA methylation levels of hypo-CHG DMRs in meiocyte (Me) versus microspore (UM) (upper) and sperm (S) relative to microspore (UM) (lower) in wild type, *cmt3a* (3a) and *cmt3b* (3b) cells. The significance was calculated with multiple comparison tests. Different letters on top of the bars indicate a significant difference (*p* < 0.05). **g** Genome Browser screen captures showing high CHG methylation sites in microspore relative to meiocyte and sperm decreased in cmt3b mutants (highlighted by grey)

Correct figure:

**Fig. 2:**
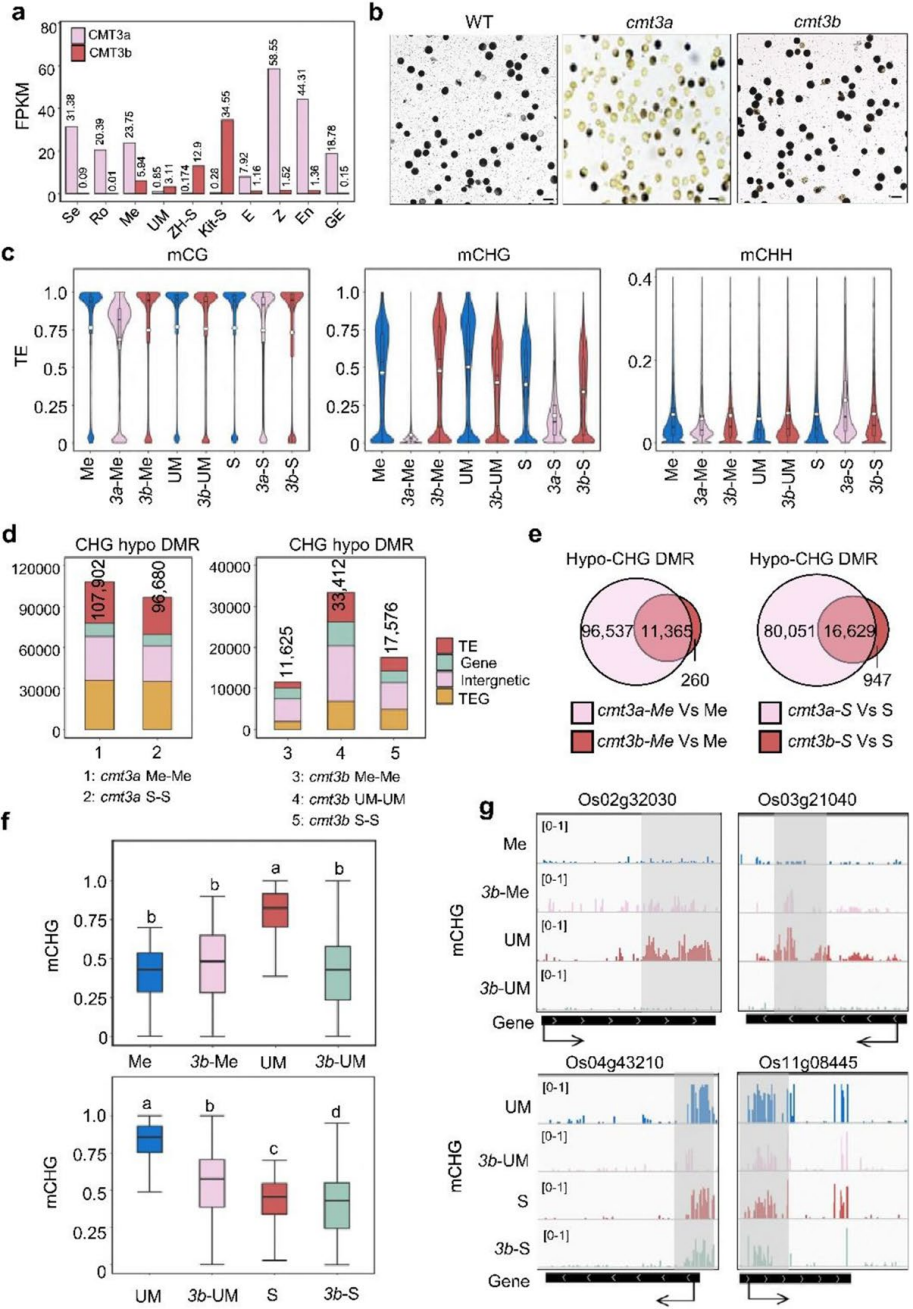
Effects of *cmt3a* and *cmt3b* mutations on DNA methylation in meiocyte, microspore and sperm. **a** Transcript levels in FPKM of rice CMT3a and CMT3b in seedling (Se), roots (Ro), meiocyte (Me), unicellular microspore (UM), sperm (S), egg (E), zygote (Z), endosperm nuclei (En, 1.5 days after fertilization) and globular embryo (GE, 3 days after fertilization) from RNA-seq data. The sperm (Kit-S) in Kitaake background was reported by Anderson et al., (2013). **b** The pollen grains of wild type and *cmt3a* and *cmt3b* mutants were I2-KI stained. Bars = 50 μm. **c** Violin plots comparing overall cytosine methylation levels of wild type and *cmt3a* and *cmt3b* mutant meiocyte (Me), unicellular microspore (UM) and sperm (S). The average methylation levels (white dots) and median values (black bars) in transposable elements (TE) are shown. Values of the methylomes are averages from the two replicates. **d** Number of differential methylated regions (DMR) in *cmt3a* and *cmt3b* relative to wild type. Relative portions in TE (> 500 bp), TEG, gene, and Intergenic regions are indicated by different colors. **e** Venn diagrams showing overlapping of hypo-CHG DMRs in *cmt3a* and *cmt3b* meiocyte (left) and sperm (right) relative to wild type cells. **f** Box plots of DNA methylation levels of hypo-CHG DMRs in meiocyte (Me) versus microspore (UM) (upper) and sperm (S) relative to microspore (UM) (lower) in wild type, *cmt3a* (3a) and *cmt3b* (3b) cells. The significance was calculated with multiple comparison tests. Different letters on top of the bars indicate a significant difference (*p* < 0.05). **g** Genome Browser screen captures showing high CHG methylation sites in microspore relative to meiocyte and sperm decreased in cmt3b mutants (highlighted by grey)
