# Supplementary material for: DNA methylation remodeling and the functional implication during male gametogenesis in rice
Source: Genome Biol. 2024 Apr 2;25:84. doi: 10.1186/s13059-024-03222-w (PMC10985897; doi:10.1186/s13059-024-03222-w)
Supplement: Supplementary file 2 — Additional file 2: Table S1. Summary of BS-seq data. [file 13059_2024_3222_MOESM2_ESM.docx]

**Additional file 2: Table S1. Summary of BS-seq data.**


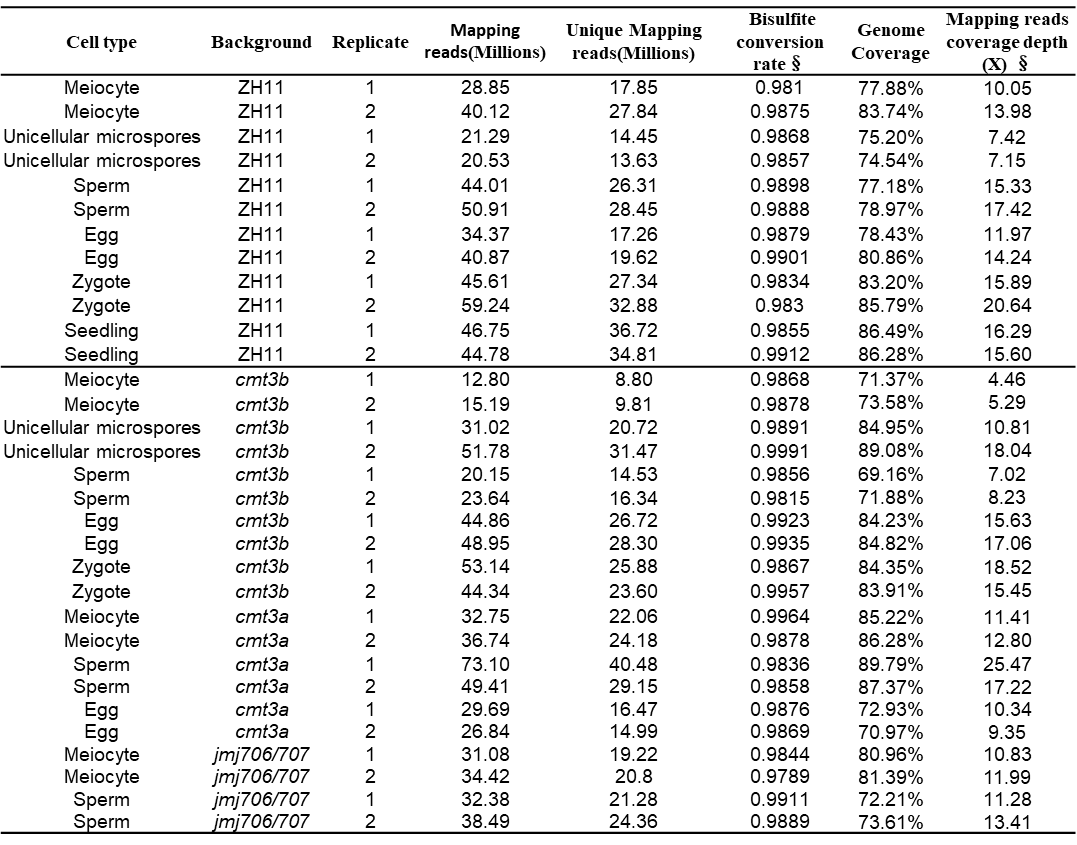


§, Bisulfite conversion rate was calculated by subtracting chloroplast DNA methylation ratio.
