# Supplementary figures and images for: DNA methylation remodeling and the functional implication during male gametogenesis in rice
Source: Genome Biol. 2024 Apr 2;25:84. doi: 10.1186/s13059-024-03222-w (PMC10985897; doi:10.1186/s13059-024-03222-w)

**Additional file 3: Table S2. Summary of RNA-seq data.**


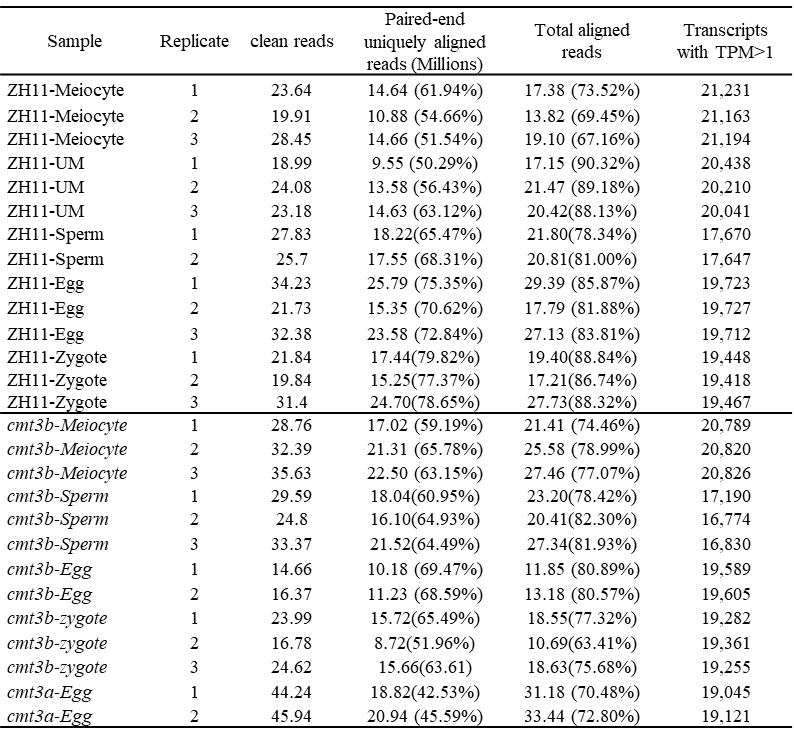

Supplement: Supplementary file 3 — Additional file 3: Table S2. Summary of RNA-seq data. [file 13059_2024_3222_MOESM3_ESM.docx]
