# Supplementary material for: DNA methylation remodeling and the functional implication during male gametogenesis in rice
Source: Genome Biol. 2024 Apr 2;25:84. doi: 10.1186/s13059-024-03222-w (PMC10985897; doi:10.1186/s13059-024-03222-w)
Supplement: Supplementary file 9 — Additional file 9. Review history. [file 13059_2024_3222_MOESM9_ESM.docx]

Review History

**First round of review**

**Reviewer 1**

**Were you able to assess all statistics in the manuscript, including the appropriateness of statistical tests used?**

Yes, and I have assessed the statistics in my report.

**Comments to author:**

In this study, the authors isolated reproductive cells of rice for bisulfite-seq and RNA-seq to investigate how DNA methylation is reprogrammed during sex cell development and the biological significance. They found that DNA methylation, particularly CHG methylation is dynamically changing during meiocyte-microspore-sperm transition. They identified the DMRs between sex cells of different stages and analyzed the genomic targets of these DMRs. To figure out which enzymes are responsible for the methylation dynamics, the authors created CMT3a/b and JMJ707/706 CRISPR mutants and isolated sex cells for bisulfite-seq and RNA-seq. By comparing methylation and gene expression between WT and the mutants, the authors conclude that CMT3b is responsible for the increased CHG methylation in microspore and that lack of CMT3a and gain of JMJ707/706 are attributed to the decreased CHG methylation in sperm. The authors also compared DNA methylation and gene expression among sperm, egg, and zygote, and proposed that CMT3b prevents the precocious expression of zygotic genes in egg. Epigenetic reprogramming during sex cell specification is a common phenomenon in plants and animals, which is important for epigenetic inheritance and sex cell development, thus being a topic under extensive study. Many advances have been made on DNA methylation reprogramming in Arabidopsis sex cells, however, whether it is conserved between dicots and monocots remains elusive. In this study, DNA methylation reprogramming in sex cells of the monocot model plant rice was investigated, which is of broad interest to epigeneticists, plant developmental biologists, and reproductive biologists. The experiments are well designed, and conclusions are largely supported by the data. But data analysis needs to improve significantly, and the results need to be discussed in a broad context.

Major concerns:

1. The authors defined DMRs as 50bp windows, which is not commonly used and less meaningful in function. DMRs need to be properly defined as regions with consecutive methylation differences between samples, which are then used for downstream analyses.

2. It is known that DNA methylation tends to occur in functional units, i.e., TEs or genes in plants. And TEs are methylated in all three sequence contexts, whereas protein-coding genes are methylated only in CG context in plants. In the manuscript, their data clearly show methylation of quite a few of protein-coding genes in all three sequence contexts, which is unusual and looks like sexual lineage-specific methylation (SLM) or sexual-lineage-hypermethylated (SLH) loci occurring in male sex cells of Arabidopsis reported previously (PMID: 29255257; PMID: 34210850). Although the methylation levels of SLM/SLH loci appear a little different among sex cells of different stages, they are generally higher in male sex cells in comparison to those in soma, hence the naming of SLM/SLH. SLM/SLH loci are two groups of loci with specific functions in gene regulation or splicing (PMID: 29255257; PMID: 34210850). Therefore, the authors should identify SLM/SLH loci genome-wide with rice data and distinguish SLM/SLH loci from those non-SLM/SLH loci according to the parameters and cutoffs defined previously (PMID: 29255257; PMID: 34210850), and perform downstream analyses separately.

3. The dynamics of mCHG levels of non-SLM/SLH loci from meiocyte to sperm should be analyzed more thoroughly. Their data show that by and large, mCHG is increased from meiocyte to microspore, then decreased from microspore to sperm. But to figure out how methylation is exactly changing, the non-SLM/SLH loci with dynamic methylation should be further categorized into 3 groups: A. loci with mCHG increased from meiocyte to microspore, then decreased in sperm; B. loci with mCHG increased from meiocyte to microspore, but not decreased in sperm; C. loci with mCHG unchanged from meiocyte to microspore, but decreased in sperm. Fully characterize the 3 groups, including the proportions, genomic targets (genes, TEs, TEGs, or intergenic regions), and genomic features (e.g., correlation with known histone modification marks, genomic locations, etc.) of individual groups.

4. The roles of CMT3b during male gametogenesis need more in-depth analyses. The CMT3b expression level in microspore is missing. Which of the 3 groups above are the targets of CMT3b for mCHG? Is CMT3b responsible for the mCHG increase in microspore and the decrease in sperm? CMT3 targets heterochromatic TEs for mCHG in Arabidopsis. Is it the case for CMT3a/b in rice sex cells? SLM/SLH loci are known to be established by RdDM activity and should be used as the control of non-SLM/SLH loci when CMT3a/b activity is analyzed.

5. In the abstract, the author claimed that in sperm, the decreased CHG methylation attributes to absence of CMT3a expression, which is not supported by the data. CMT3a mutation abolishes mCHG in all types of sex cells not just in sperm, whereas CMT3b mutation reduced mCHG in microspore to the level of sperm, suggesting CMT3b expression changes might be responsible for the decreased CHG methylation in sperm. The authors should check if CMT3b is downregulated from microspore to sperm.

6. JMJ706/707 expression levels in microspore are missing. Like what should be done for CMT3b, the roles of JMJ706/707 in regulating SLM/SLH and non-SLM/SLH loci should be studied. Moreover, the target loci of JMJ706/707 and CMT3a/b should be compared.

Minor concerns:

1. In Fig. 2A, 3a-S has a small portion of mCHG retained, please do some analyses on these loci to reveal which enzymes/pathways might be responsible for it, e.g., CMT3b, canonical RdDM, or sex cell-specific RdDM?

2. Line 27 on Page 10, "Among the upregulated genes, 416 overlapped with previously identified genes expressed in rice zygotes (Figure 6B).". What is the logic of only the 416 overlapped genes being used for downstream analyses?

3. Fig. S4A shows no expression of CMT3b in egg. How did the CMT3b mutation make differences on mCHG levels and gene expression in egg?

4. Please describe how TEGs and DMR-targeted genes are defined in Methods.

5. Please compare the methylomes of sperm, egg, and zygote. Is zygote methylome the add-up of sperm and egg's methylomes?

6. Multiple comparisons should be done instead of t test for Fig. 2D, 3C.

7. Please provide in a table with the quality control of the libraries used in the study including conversion rates and coverage, etc.

**Reviewer 2**

**Were you able to assess all statistics in the manuscript, including the appropriateness of statistical tests used?**

Yes, and I have assessed the statistics in my report.

**Comments to author:**

Xue Li and colleagues present a manuscript in which they address whether DNA methylation reprogramming occurs within the germline of rice. This is an interesting question, as methylation states are shown to be thoroughly reprogrammed in mammals, but most work in plant systems (primarily Arabidopsis) has pointed towards very limited reprogramming.

The manuscript is very clearly written with visually clear and well thought out figures. However, I have several major disagreements with the main conclusions and framing of the paper based on the data presented. In my opinion, there are possible valuable advancements to the field in the work on CMT3 and JMJ mutants, but this work should not necessarily be framed around the concept of reprogramming, which the authors do not show. It's also possible that there is some interesting biology uncovered by the RNA-seq, but these are given less attention than the DNA methylation data. Lastly, the DMR calling in this paper is hard to interpret as the authors have chosen methods that identify a very large number of regions. This is addressable with revisions to the analysis methods. The authors should also demonstrate as comprehensively as possible that their "low input" method is generating sufficiently high depth and quality data.

Major concerns:

- In Figure 1A, the authors present overall CG, CHG and CHH methylation levels in meiocytes, microspores and sperm cells. They point to reduced CHG and CHH levels in sperm as an interesting observation. However, Fig. 1A shows that this decrease is a small effect - the average methylation levels look to be about 5-10% lower in sperm than microspores. What this means in terms of biology, is that 5-10% of individual cells may lose methylation at any given CHG site, which is a long way off what could be considered "reprogramming". It is worth noting at this point that it is well known that during male germline development there is a cell cycle arrest that could account for smaller fluctuations in DNA methylation levels. This cell cycle effect is discussed in detail in this paper: Borges et al, Current Biology. 31, 591-600.

- I am also highly concerned about how the authors have called DMRs. DMR calling can very easily yield a lot of false positives, and the extremely large number of DMRs (>100,000 for CHH!) called is questionable. Part of this may be due to calling very short windows (50bp), which should be merged if they are adjacent to each other to provide a more realistic estimate of the number of differentially methylated loci.

- The authors DMR calling method is new to me and does not seem to be very consistent with "industry standard" methods used by most people in the field (which are also not infallible). The main problem is that their windows are so small that each window could have average methylation levels strongly affected by just one or two cytosines. Additionally, they pool depth across all cytosines, so the window average could feasibly be affected by just one high depth cytosine. I think the authors need to add an additional filter such that there are a minimum number of cytosines covered by 5 or more reads within the window, as it typical in most DMR calling methods. I also think that the windows should be larger so that a greater number of cytosines can contribute to the average methylation levels for the window.

- The authors present bisulfite sequencing data from a method that allows them to generate data from a small number of cells. Bisulfite sequencing can be very difficult from a small number of cells, so the authors should present statistics on median, mean and interquartile read depth for their samples. If they have truly managed to get decent read coverage from a small number of cells this is impressive (the browser snapshots look promising) but they must show convincing data of genomic coverage for readers to know the data is reliable.

Minor concerns

- The authors DMR method needs to be reworded in the results section, as it initially reads as though the authors selected 50bp windows with 20 or more cytosines within that sequence. Upon reading the methods it is clear they meant 20 or more reads covering all Cs, which makes more sense.

- The violin plots for methylation level should have their y axis capped at 1.0, as methylation cannot be higher than 1.0.

- Figure 2D, the 3 cell types can all be presented together in one figure, rather than two panels with 2 cell types each

- Figure 2D the y axis label is missing

- I think the CMT3 expression data in Supp. Fig. 4A and C are interesting and would be beneficial to include in Figure 2. They add to the story as CMT3b is expressed in reproductive tissues.

**Authors Response**

**Point-by-point responses to the reviewers’ comments:**

Reviewer #1：

In this study, the authors isolated reproductive cells of rice for bisulfite-seq and RNA-seq to investigate how DNA methylation is reprogrammed during sex cell development and the biological significance. They found that DNA methylation, particularly CHG methylation is dynamically changing during meiocyte-microspore-sperm transition. They identified the DMRs between sex cells of different stages and analyzed the genomic targets of these DMRs. To figure out which enzymes are responsible for the methylation dynamics, the authors created CMT3a/b and JMJ707/706 CRISPR mutants and isolated sex cells for bisulfite-seq and RNA-seq. By comparing methylation and gene expression between WT and the mutants, the authors conclude that CMT3b is responsible for the increased CHG methylation in microspore and that lack of CMT3a and gain of JMJ707/706 are attributed to the decreased CHG methylation in sperm. The authors also compared DNA methylation and gene expression among
sperm, egg, and zygote, and proposed that CMT3b prevents the precocious expression of zygotic genes in egg. Epigenetic reprogramming during sex cell specification is a common phenomenon in plants and animals, which is important for epigenetic inheritance and sex cell development, thus being a topic under extensive study. Many advances have been made on DNA methylation reprogramming in Arabidopsis sex cells, however, whether it is conserved between dicots and monocots remains elusive. In this study, DNA methylation reprogramming in sex cells of the monocot model plant rice was investigated, which is of broad interest to epigeneticists, plant developmental biologists, and reproductive biologists. The experiments are well designed, and conclusions are largely supported by the data. But data analysis needs to improve significantly, and the results need to be discussed in a broad context.

Major concerns

1. The authors defined DMRs as 50bp windows, which is not commonly used and less meaningful in function. DMRs need to be properly defined as regions with consecutive methylation differences between samples, which are then used for downstream analyses.

*REPLY: Thanks for the comments. Following the advice, we have recalculated DMRs in 100 bp windows with criteria routinely used for analysis of rice tissue methylomes. The detail of DMR calling is described in Methods.*

*Because the methylone data were obtained from low cell number sample (not bulk tissues), we had used 50 bp bins to call DMRs as in other published rice sex cell methylome papers.*

2. It is known that DNA methylation tends to occur in functional units, i.e., TEs or genes in plants. And TEs are methylated in all three sequence contexts, whereas protein-coding genes are methylated only in CG context in plants. In the manuscript, their data clearly show methylation of quite a few of protein-coding genes in all three sequence contexts, which is unusual and looks like sexual lineage-specific methylation (SLM) or sexual-lineage-hypermethylated (SLH) loci occurring in male sex cells of Arabidopsis reported previously (PMID: 29255257; PMID: 34210850). Although the methylation levels of SLM/SLH loci appear a little different among sex cells of different stages, they are generally higher in male sex cells in comparison to those in soma, hence the naming of SLM/SLH. SLM/SLH loci are two groups of loci with specific functions in gene regulation or splicing (PMID: 29255257; PMID: 34210850). Therefore, the authors should identify SLM/SLH loci genome-wide with rice data and distinguish SLM/SLH loci from those non-SLM/SLH loci according to the parameters and cutoffs defined previously (PMID: 29255257; PMID: 34210850), and perform downstream analyses separately.

*Reply: Thanks for the comments and the suggestions. Compared with Arabidopsis, rice genome DNA methylation displays slight differences in methylation levels and genomic distribution. For instance, rice mCHH is enriched at euchromatin regions and many genes are found to be methylated at CHH sites (ref 4, 5), especially around TSS. There are also quite a number of genes with mCHG related to allelic-specific expression in rice heterosis (ref 6). We have added the sentence to Background.*

*We have performed analysis of sexual lineage methylation in rice cells as advised. With the published method used in Arabidopsis SLH/SLM identification, we have identified 555 SLH in rice meiocyte, microspore and sperm cells. We found that cmt3b mutation reduced SLH levels mainly in microspore (As the cmt3a mutation erases nearly all mCHG in any cell, a specific effect of the mutation on SLH cannot be discriminated). By contrast, the jmj706/7 had no clear effect on SLH in the cells. The data are shown in* *Additional file 1: Fig. S9 and commented in the MS text.*

*From the 555 SLH, we could divide into 215 SLM and 340 canonical SLH. Most of the canonical SLH are mapped to TE, while SLM are mainly mapped to genes, as observed in Arabidopsis. We found 132 genes (enriched for translational function) are targeted by SLM, which showed lower expression in sperm compared to soma (seedling) or meiocyte/microspore and the cmt3b mutation resulted in higher expression of some of the genes in sperm. The data are shown in Additional file 1: Fig. S10 and commented in the MS text.*

3. The dynamics of mCHG levels of non-SLM/SLH loci from meiocyte to sperm should be analyzed more thoroughly. Their data show that by and large, mCHG is increased from meiocyte to microspore, then decreased from microspore to sperm. But to figure out how methylation is exactly changing, the non-SLM/SLH loci with dynamic methylation should be further categorized into 3 groups: A. loci with mCHG increased from meiocyte to microspore, then decreased in sperm; B. loci with mCHG increased from meiocyte to microspore, but not decreased in sperm; C. loci with mCHG unchanged from meiocyte to microspore, but decreased in sperm. Fully characterize the 3 groups, including the proportions, genomic targets (genes, TEs, TEGs, or intergenic regions), and genomic features (e.g., correlation with known histone modification marks, genomic locations, etc.) of individual groups.

*REPLY: Thanks for the suggestion. We have performed the advised analysis and divided the methylation loci into 3 clusters: Cluster A (with mCHG increased from meiocyte to microspore, then decreased in sperm) and cluster B (with mCHG increased from meiocyte to microspore, but not decreased in sperm) loci mainly correspond to gene or genic regions. Cluster C (with mCHG unchanged from meiocyte to microspore, but decreased in sperm) are enriched for TE and TEG (Additional file 1: Fig. S3a, b).*

*Cluster A and B (especially cluster A) are enriched for active marks (H3K36me3, K27me3, H3K9ac), Cluster C is enriched for H3K9me2 (Additional file 1: Fig. S3d).*

*The data are commented in the MS text.*

4. The roles of CMT3b during male gametogenesis need more in-depth analyses. The CMT3b expression level in microspore is missing. Which of the 3 groups above are the targets of CMT3b for mCHG? Is CMT3b responsible for the mCHG increase in microspore and the decrease in sperm? CMT3 targets heterochromatic TEs for mCHG in Arabidopsis. Is it the case for CMT3a/b in rice sex cells? SLM/SLH loci are known to be established by RdDM activity and should be used as the control of non-SLM/SLH loci when CMT3a/b activity is analyzed.

*REPLY: Thanks for the suggestion. We have added the CMT3b microspore expression data in Fig. 2a. We found that in microspore cmt3b mutation reduced the mCHG levels of all three clusters in microspore and reduced mCHG levels of Cluster A and B in sperm (Additional file 1: Fig. S3c). As CMT3b is expressed in microspore and the cmt3b mutation almost erased the increased mCHG in microspore, we suggest that CMT3b is responsible the increase of mCHG in microspore. Because CMT3b expression was detected in sperm (Fig. 2a), it is unlikely the decrease of mCHG in sperm is related to CMT3b. The present data showing a large effect of the cmt3b mutation for cluster A and B in both microspore and sperm, suggest that CMT3b may have a preference for mCHG in genic regions (cluster A and B) in rice sex cells. As replied above, the cmt3a mutation erases almost all mCHG, its effect on a specific cluster cannot be demonstrated. As the cmt3a and cmt3b mutations largely reduced mCHG in TEs (Fig. 2c), CMT3a/b also mainly target TEs for mCHG in rice as in Arabidopsis. As cmt3b mutation reduced SLH, we think that besides RdDM, CMT3 is also involved in SLH, at least in rice. Perhaps, CMT3 is required to maintain SLH/SLM initially mediated by RdDM (Additional file 1: Fig. S9b).*

*The new data are commented in the revised MS text.*

5. In the abstract, the author claimed that in sperm, the decreased CHG methylation attributes to absence of CMT3a expression, which is not supported by the data. CMT3a mutation abolishes mCHG in all types of sex cells not just in sperm, whereas CMT3b mutation reduced mCHG in microspore to the level of sperm, suggesting CMT3b expression changes might be responsible for the decreased CHG methylation in sperm. The authors should check if CMT3b is downregulated from microspore to sperm.

*REPLY: Thanks for point. We have revised Abstract regarding to the statement. The present data suggested that CMT3b is required for the increase of mCHG in microspore. As advised, we have checked* *CMT3b expression in sperm, which was at a high level than in microspore (Fig. 2a). It is unlikely that CMT3b is responsible for the decrease of mCHG in sperm.*

6. JMJ706/707 expression levels in microspore are missing. Like what should be done for CMT3b, the roles of JMJ706/707 in regulating SLM/SLH and non-SLM/SLH loci should be studied. Moreover, the target loci of JMJ706/707 and CMT3a/b should be compared.

*REPLY: We have added the expression profile of JMJ706/7 in microspore in Additional file 1: Fig. S7a. As shown in Additional file 1: Fig. S9b, there seems no effect of the jmj706/7 mutations on SLH. For non-SLH loci, jmj706/7 mutations led to increased mCHG of cluster B in meiocyte and of cluster C in sperm (Additional file 1: Fig. S3c). Because cluster A and B are enriched for genic and intergenic regions while cluster C is enriched for TE and TEGs, the effect of jmj706/7 on non-SLH loci suggest that JMJ706/7 controls different genomic regions during male gametogenesis. There is no clear overlap between the target loci of JMJ706/7 and CMT3b (Additional file 1: Fig. S8b), but about ½ to 2/3 of the JMJ706/7 targets overlapped with those of CMT3a, which is due to the overwhelming effect of cmt3a mutation effect on mCHG in the genome.*

Minor concerns

1. In Fig. 2A, 3a-S has a small portion of mCHG retained, please do some analyses on these loci to reveal which enzymes/pathways might be responsible for it, e.g., CMT3b, canonical RdDM, or sex cell-specific RdDM?

*REPLY: Thanks for the point. As DRM2 and CMT2 show high expression in sperm (Additional file 1: Fig. S6a) and cmt3b had no clear effect on mCHG loci retained in cmt3a sperm (Additional file 1: Fig. S6b). The remaining mCHG in cmt3a sperm may be due to RdDM or CMT2. This is supported by the observation that the drm2/cmt2 mutations reduced the mCHG levels of these loci in leaves (Additional file 1: Fig. S6b).*

*Comparison between SLH loci and mCHG regions in cmt3a sperm revealed no overlap (see below). It is unlikely that a sexual specific RdDM is involved in the remaining mCHG in cmt3a sperm.*

2. Line 27 on Page 10, "Among the upregulated genes, 416 overlapped with previously identified genes expressed in rice zygotes (Figure 6B).". What is the logic of only the 416 overlapped genes being used for downstream analyses?

*REPLY: Due to difficulties to isolated zygotes without contamination of other maternal cells, zygotic transcriptomes may contain non-zygotic transcripts. By merging several zygotic transcriptomes obtained from different laboratories should help to ascertain that the genes to be analyzed were true zygotic genes.*

3. Fig. S4A shows no expression of CMT3b in egg. How did the CMT3b mutation make differences on mCHG levels and gene expression in egg?

*REPLY: With log2 of TPM, the values appeared negative as shown in previous version of the supplemental Figure 4A. Although very low, CMT3b expression can be detected in egg and zygote, when displayed with FPKM in the revised Fig. 2a.*

4. Please describe how TEGs and DMR-targeted genes are defined in Methods.

*REPLY: We have added the description and related reference in the methods section.*

5. Please compare the methylomes of sperm, egg, and zygote. Is zygote methylome the add-up of sperm and egg's methylomes?

*REPLY: No, we have previously shown that the rice zygotic methylation is not the add-up of sperm and egg methylomes (ref 17, 19).*

6. Multiple comparisons should be done instead of t test for Fig. 2D, 3C.

*REPLY: We have changed to multiple comparisons.*

7. Please provide in a table with the quality control of the libraries used in the study including conversion rates and coverage, etc

*REPLY: We have revised Additional file 2: Table S1 as advised. Coverage (with reads= and >1, about 70-90%) and conversion (>98%) rates are added to the table.*

REVIEWER 2

Xue Li and colleagues present a manuscript in which they address whether DNA methylation reprogramming occurs within the germline of rice. This is an interesting question, as methylation states are shown to be thoroughly reprogrammed in mammals, but most work in plant systems (primarily Arabidopsis) has pointed towards very limited reprogramming. The manuscript is very clearly written with visually clear and well thought out figures. However, I have several major disagreements with the main conclusions and framing of the paper based on the data presented.

In my opinion, there are possible valuable advancements to the field in the work on CMT3 and JMJ mutants, but this work should not necessarily be framed around the concept of reprogramming, which the authors do not show.

It's also possible that there is some interesting biology uncovered by the RNA-seq, but these are given less attention than the DNA methylation data.

Lastly, the DMR calling in this paper is hard to interpret as the authors have chosen methods that identify a very large number of regions. This is addressable with revisions to the analysis methods. The authors should also demonstrate as comprehensively as possible that their "low input" method is generating sufficiently high depth and quality data.

Major concerns

1. In Figure 1A, the authors present overall CG, CHG and CHH methylation levels in meiocytes, microspores and sperm cells. They point to reduced CHG and CHH levels in sperm as an interesting observation. However, Fig. 1A shows that this decrease is a small effect - the average methylation levels look to be about 5-10% lower in sperm than microspores. What this means in terms of biology, is that 5-10% of individual cells may lose methylation at any given CHG site, which is a long way off what could be considered "reprogramming". It is worth noting at this point that it is well known that during male germline development there is a cell cycle arrest that could account for smaller fluctuations in DNA methylation levels. This cell cycle effect is discussed in detail in this paper: Borges et al, Current Biology. 31, 591-600.

*REPLY: Thanks for comments. Violin plots in Fig. 1a are mass data comparisons of genome-wide overall sequenced methylation levels between different samples. Rather, density plots (pair-wise comparison of the same bins between two samples) in Fig. 1b allow estimating the difference of methylation levels at the same genomic regions between the compared samples. It can be seen that there is a clear shift of mCHG from 0 (no difference) to -1 between sperm and microspore. We agree that cell cycle arrest in sperm may account for fluctuations in DNA methylation levels in sperm. We have discussed the point and cited the reference: Borges et al, Current Biology. 31, 591-600.*

*Still, high mCHG levels in microspore at several genes/loci appeared totally erased in sperm (shown in Additional file 1: Fig. S3f). If cell cycle arrest at S phase prevented CMT3 to maintain mCHG in newly synthesized DAN strand, there should have been some mCHG levels at these loci (on the old DNA strand) in sperm.*

2. I am also highly concerned about how the authors have called DMRs. DMR calling can very easily yield a lot of false positives, and the extremely large number of DMRs (>100,000 for CHH!) called is questionable. Part of this may be due to calling very short windows (50bp), which should be merged if they are adjacent to each other to provide a more realistic estimate of the number of differentially methylated loci.

*REPLY: Thanks for the comment and advice. We have changed DMR calling parameters by using 100 bp bins and merged neighboring DMRs as suggested and thoroughly revised the text. The new DMR numbers show similar trends as previous ones. The details are described in methods section.*

3. The authors DMR calling method is new to me and does not seem to be very consistent with "industry standard" methods used by most people in the field (which are also not infallible). The main problem is that their windows are so small that each window could have average methylation levels strongly affected by just one or two cytosines. Additionally, they pool depth across all cytosines, so the window average could feasibly be affected by just one high depth cytosine. I think the authors need to add an additional filter such that there are a minimum number of cytosines covered by 5 or more reads within the window, as it typical in most DMR calling methods. I also think that the windows should be larger so that a greater number of cytosines can contribute to the average methylation levels for the window.

*REPLY: Thanks for the advice. We have used the advised methods to call DMRs:* *To avoid methylation being strongly influenced by single cytosine sites, the methylation level of each cytosine is calculated separately and then averaged for all cytosines to represent the methylation level for each bin (bin methylation level = (sum of individual cytosine methylations) / (number of cytosines within 100 bp).*

*The following sentences are added to the methods section:*

*“To identify differential methylated regions (DMRs), the whole genome was divided into 100-bp bins. Bins that contained at least five cytosines each and every cytosine with at least a five-fold coverage were retained, absolute methylation difference of 0.5, 0.3, and 0.1 for CG, CHG, and CHH, respectively, and P values < 0.01 (Fisher’s exact test) were considered as DMRs. Neighboring DMRs within 200 bp were merged.”*

4. The authors present bisulfite sequencing data from a method that allows them to generate data from a small number of cells. Bisulfite sequencing can be very difficult from a small number of cells, so the authors should present statistics on median, mean and interquartile read depth for their samples. If they have truly managed to get decent read coverage from a small number of cells this is impressive (the browser snapshots look promising) but they must show convincing data of genomic coverage for readers to know the data is reliable.

*REPLY: Thanks for the comments.*

*Genomic coverage (with reads= and >1, about 70-90%) and conversion (>98%) rates are added to Additional file 2: Table S1, which are close to BS-seq data from bulk tissues (i.e. seedlings). We have performed boxplot analysis of the mapped reads, coverage depth and genome coverage at different read numbers (please see Additional file 1: Fig. S1c). The present data are comparable to or better than the data previously published in rice sex cells (ref 17).*

Minor concerns

1. The authors DMR method needs to be reworded in the results section, as it initially reads as though the authors selected 50bp windows with 20 or more cytosines within that sequence. Upon reading the methods it is clear they meant 20 or more reads covering all Cs, which makes more sense.

*REPLY: we have deleted wording (20 informative cytosines) from the Results section. The details of DMR calling are revised in Methods.*

2. The violin plots for methylation level should have their y axis capped at 1.0, as methylation cannot be higher than 1.0.

*REPLY: We have corrected the y-axis.*

3. Figure 2D, the 3 cell types can all be presented together in one figure, rather than two panels with 2 cell types each

*REPLY: Because the compared loci in upper (hyper methylated in microspore versus meiocyte) and lower (hypomethylated in sperm versus microspore) parts of Fig. 2f (previous Fig 2D) are not the same, putting them together would be confusing.*

4.Figure 2D the y axis label is missing

*REPLY: y-axis added.*

5. I think the CMT3 expression data in Supp. Fig. 4A and C are interesting and would be beneficial to include in Figure 2. They add to the story as CMT3b is expressed in reproductive tissues.

*REPLY: Thanks for the suggestion. We have added the previous Supp. Fig. 4A to Figure 2.*

**Second round of review**

**Reviewer 1**

The authors have performed substantial analyses as requested and addressed most of my concerns. One piece of the new data in Fig. S3f shows potentially very interesting phenomenon. The methylation of the genes shown in Fig. 3f exhibits very similar patterns to RNA-seq signals, i.e., enriched in exons. If true, it suggests a special methylase activity targeting exons only exists in microspore. The authors should 1) confirm it is not an artifact, e.g., contaminated reads from mRNA-seq in multiplexed NGS sequencing of BS-seq and mRNA-seq; 2) perform more analyses to show the correlation of methylation of the 315 genes with exons, using introns as the control, and to analyze if CMT3b is possibly responsible for such seemingly exon-specific methylation; 3) provide additional tracks of CG and CHH contexts and those of 3a-UM and 3b-UM in Fig. 3f.

**Reviewer 2**

I have reviewed the revisions conducted by the authors and I am satisfied that the authors have corrected the issues with analysis and interpretation identified in my previous review and by the other reviewer. In particular I found the new mapping statistics in Figure S1 to be convincing, as this is quite good performance for a low input experiment. I would now be happy if this article proceeded to publication.

**Authors Response**

**Point-by-point responses to the reviewers’ comments:**

Reviewer #1:

The authors have performed substantial analyses as requested and addressed most of my concerns. One piece of the new data in Fig. S3f shows potentially very interesting phenomenon. The methylation of the genes shown in Fig. s3f exhibits very similar patterns to RNA-seq signals, i.e., enriched in exons. If true, it suggests a special methylase activity targeting exons only exists in microspore. The authors should 1) confirm it is not an artifact, e.g., contaminated reads from mRNA-seq in multiplexed NGS sequencing of BS-seq and mRNA-seq; 2) perform more analyses to show the correlation of methylation of the 315 genes with exons, using introns as the control, and to analyze if CMT3b is possibly responsible for such seemingly exon-specific methylation; 3) provide additional tracks of CG and CHH contexts and those of 3a-UM and 3b-UM in Fig. s3f.

*REPLY: Thank you for your suggestion.*

*1) The BS-seq and the RNA-seq samples were sequenced separately. There should be no any interference between the two libraries;*

*2) We have analyzed the mCHG levels in the 315 genes from cluster A. These genes showed indeed higher mCHG levels in exons than in introns (Additional file 1: Fig. S4b). The cmt3b mutation reduced the mCHG levels from both exons and introns in microspore (Additional file 1: Fig. S4a, b). In conclusion, CMT3b may preferentially target gene exons for mCHG. We have commented the data in results section.*

*3) Screenshots of the related genes mCG and mCHH are added (Additional file 1: Fig. S4a). No cmt3a-UM mutant data is currently available.*

**Third round of review**

**Reviewer 1**

The authors have addressed all my concerns.
